# Integrating Network Analysis and Metabolomics to Reveal Mechanism of Huaganjian Decoction in Treatment of Cholestatic Hepatic Injury

**DOI:** 10.3389/fphar.2021.773957

**Published:** 2022-01-19

**Authors:** Qin Dong, Jiao Chen, Yan-Ping Jiang, Zong-Ping Zhu, Yong-Feng Zheng, Jin-Ming Zhang, Zhen Zhang, Wen-Qing Chen, Shi-Yi Sun, Lan Pang, Xin Yan, Wan Liao, Chao-Mei Fu

**Affiliations:** ^1^ State Key Laboratory of Southwestern Chinese Medicine Resources, Pharmacy College, Chengdu University of Traditional Chinese Medicine, Chengdu, China; ^2^ Department of Biology, Hong Kong Baptist University, Hong Kong, China; ^3^ Chengdu Institute of Chinese Herbal Medicine, Chengdu, China

**Keywords:** huaganjian decoction, cholestatic hepatic injury, metabolomics, serum pharmacochemistry, network analysis

## Abstract

Huaganjian decoction (HGJD) was first recorded in the classic “*Jing Yue Quan Shu*” during the Ming dynasty, and it has been extensively applied in clinical practice to treat liver diseases for over 300 years in China. However, its bioactive constituents and relevant pharmacological mechanism are still unclear. In this study, a strategy integrating network analysis and metabolomics was applied to reveal mechanism of HGJD in treating cholestatic hepatic injury (CHI). Firstly, we observed the therapeutic effect of HGJD against CHI with an alpha-naphthylisothiocyanate (ANIT) induced CHI rat model. Then, we utilized UPLC-Q-Exactive MS/MS method to analyze the serum migrant compounds of HGJD in CHI rats. Based on these compounds, network analysis was conducted to screen for potential active components, and key signaling pathways interrelated to therapeutic effect of HGJD. Meanwhile, serum metabolomics was utilized to investigate the underlying metabolic mechanism of HGJD against CHI. Finally, the predicted key pathway was verified by western blot and biochemical analysis using rat liver tissue from *in vivo* efficacy experiment. Our results showed that HGJD significantly alleviated ANIT induced CHI. Totally, 31 compounds originated from HGJD have been identified in the serum sample. PI3K/Akt/Nrf2 signaling pathway related to GSH synthesis was demonstrated as one of the major pathways interrelated to therapeutic effect of HGJD against CHI. This research supplied a helpful strategy to determine the potential bioactive compounds and mechanism of traditional Chinese medicine.

## 1 Introduction

Cholestatic hepatic injury (CHI) occurs mainly due to intrahepatic cholestasis. Its main characteristics are aberrant metabolism of bile acid and accumulating of toxic bile acids in the liver. Signals such as oxidative stress and inflammatory responses are activated, leading to parenchymal cell death of liver and bile duct (Cai and Boyer, 2021). Without proper treatment, CHI may further develop into fibrosis, cirrhosis, hepatocellular carcinoma and eventually liver failure (Pollock and Minuk, 2017). Currently, ursodeoxycholic acid (UDCA), and obeticholic acid (OCA) are main therapeutic options for CHI (Hohenester and Beuers, 2017). However, some patients have an inadequate response to UDCA therapy and the side effects of OCA in this treatment remain to be improved (Hirschfield et al., 2015; Kowdley et al., 2018).

In recent years, with rapid preclinical and clinical research of traditional Chinese medicine (TCM), TCM has shown advantage in clinical practice to treat CHI. Huaganjian decoction (HGJD) is a classic Chinese medicine formula, which is first recorded in the classic “*Jing Yue Quan Shu*” during the Ming dynasty. This formula is composed of 7 botanical drugs: Citri Reticulatae Pericarpium Viride (*Citrus reticulata* Blanco, Qingpi, 7.5 g), Gardeniae Fructus (*Gardenia jasminoides* Ellis, Zhizi, 5.6 g), Paeoniae Radix Alba (*Paeonia lactiflora* Pall., Baishao, 7.5 g), Moutan Cortex (*Paeonia suffruticosa* Andr., Mudanpi, 5.6 g), Alismatis Rhizaoma (*Alisma plantago-aquatica* Linn., Zexie, 5.6 g), Fritillariae Thunbergii Bulbus (*Fritillaria thunbergii* Miq., Zhebeimu, 11.2 g), and Citri Reticulatae Pericarpium (*Citrus reticulata* Blanco., Chenpi, 7.5 g). It is characterized by comprehensive therapy, which can relieve stagnation of liver qi, extenuate liver fire, and invigorate liver blood. It was widely used in the clinical practice to protect against liver injury (He, 2009; Liu L F et al., 2019; Li, 2021). Moreover, HGJD has been listed as one of the 100 ancient classic prescriptions highly valued by the Chinese government. According to modern medical research, HGJD could promote bile excretion, protect liver cells, and inhibit the proliferation of activated hepatic stellate cells HSC-T6 (Gao et al., 2019). Qingpi was the principle drug of HGJD, which could protect against liver damage caused by carbon tetrachloride and promote bile excretion (Jin et al., 2007). Zhizi was the minister drug of HGJD, which could attenuate live cell injury and fibrosis as demonstrated in both animal and human studies (Chen et al., 2012). However, the specific mechanism of HGJD protecting against CHI is still not revealed. Besides, the bioactive compounds that contribute to its therapeutic efficacy remain unclear.

**FIGURE 1:**
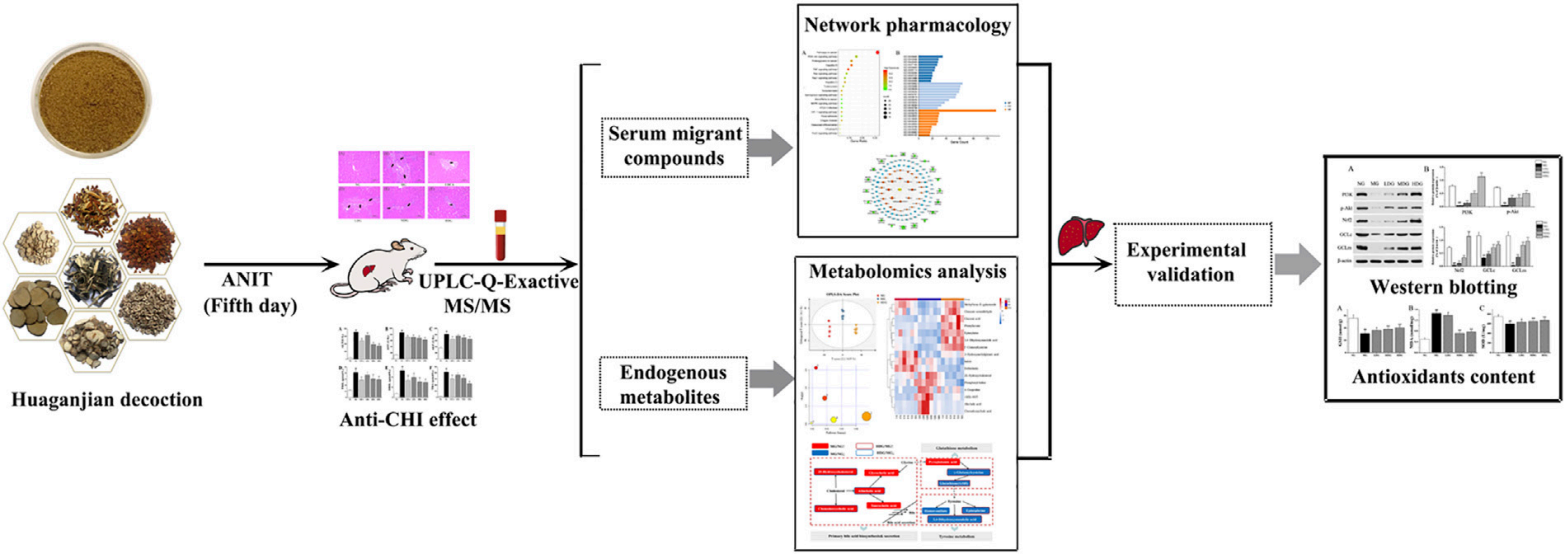
The graphical abstract of this study.

In this research, a comprehensive method integrating network analysis and metabolomics was used to illustrate mechanism of HGJD in treating CHI. Firstly, we observed the therapeutic effect of HGJD against CHI by evaluating the serum biochemical indices and histopathology of liver with an ANIT induced CHI rat model. Then, we utilized UPLC-Q-Exactive MS/MS method to analyze the serum migrant compounds of HGJD in ANIT induced CHI rat model. Network analysis based on serum migrant compounds of HGJD was used to explore the correlations among potential active ingredients, targets and signaling pathways interrelated to therapeutic effect of HGJD. Meanwhile, non-target serum metabolomics were applied to investigate the underlying metabolic mechanism of HGJD against CHI. Finally, we further validated the predicted pathway by western blot and biochemical analysis using rat liver tissue from *in vivo* efficacy experiment. This research would offer experimental basis for further research on HGJD in treatment of CHI, and offer a novel insight into improving the treatment for CHI. A graphical abstract of this study is presented in Figure 1.

## 2 Materials and Methods

### 2.1 Chemicals and Reagents

All 22 standard compounds used in this study (synephrine, gallic acid, chlorogenic acid, geniposide, peimine, peiminine, albiflorin, paeoniflorin, rutin, narirutin, crocin, naringin, hesperidin, neohesperidin, didymin, quercetin, benzoylpaeoniflorin, paeonol, sinensetin, nobiletin, hepta-3, and alisol B 23-acetate) were supplied by Weikeqi Biotechnology Co., Ltd (Chengdu, China), and the purity were all greater than 98%. UDCA administrated as positive control was supplied by Shanghai Aladdin Biochemical Co., Ltd. (Shanghai, China). ANIT and medical-grade soybean oil were purchased from Shanghai Aladdin Biochemical Co., Ltd. (Shanghai, China). β-actin was obtained from Wuhan Servicebio Technology Co., Ltd. (Wuhan, China). Antibody PI3K was purchased from Beijing Boaosen Biotechnology Co., Ltd. (Beijing, China). Antibody p-AKT was bought from Affinity Biosciences Ltd. (Jiangsu, China). Antibody Nrf2 was purchased from Wuhan Sanying Biotechnology Co., Ltd. (Wuhan, China). Antibody GCLc and GCLm were obtained from abcam (Cambridge, United Kingdom). Commercially available kits were provided by Nanjing Jian cheng Institute of Biotechnology (Nanjing, China) and Changchun Huili Biological Technology Co., Ltd. (Changchun, China). All other chemicals and reagents were belonged to analytical grade, and were purchased from commercial sources.

All botanical drugs in the HGJD formula were bought from Sichuan Neautus TCM Co., Ltd (Chengdu, China) and identified by Professor Guihua Jiang of the School of Pharmacy, Chengdu University of Traditional Chinese Medicine.

### 2.2 Preparation of HGJD Samples

The botanical drug mixture of Citri Reticulatae Pericarpium Viride, Gardeniae Fructus, Paeoniae Radix Alba, Moutan Cortex, Alismatis Rhizaoma, Fritillariae Thunbergii Bulbus, and Citri Reticulatae Pericarpium (4: 3: 4: 3: 3: 6: 4) was soaked with 9 vol. distilled water for 30 min and maintained boiling for 25 min. The extraction solution was filtered and evaporated under reduced pressure to the weight of the original mixture. Finally, freeze drying was carried out for concentrated extract to obtain lyophilized powder. The lyophilized powder yield was about 33.12%. The lyophilized powder was stored in different airtight packages before chemical and pharmacological studies. Quality control of the sample was led according to our previous research (Nie et al., 2020). Moreover, UPLC-Q-Exactive MS/MS was used to analyze the chemical composition of HGJD sample (Supplementary Figure S1; Supplementary Table S1). Every day before administration, lyophilized powder of HGJD was dissolved in different volumes of water to prepare low (0.15 g/ml), medium (0.30 g/ml) and high dosage (0.60 g/ml) samples.

### 2.3 Animals

SPF Male Sprague-Dawley (SD) rats (220 ± 20 g) were purchased from Beijing Sibefu Biotechnology Co., Ltd. (Beijing, China) (Certificate No. SCXK (Jing) 2019–0010). Animal Ethics Committee of Chengdu University of TCM (Chengdu, China) approved this experiment, and international rules for care and use of laboratory animals were strictly followed. The room temperature was regulated at 20 ± 2 °C with 50 ± 20% humidity and equipped with a 12 h light/dark cycle. We allowed the rats to eat pure water and food freely. All animals were adapted to the conditions for 1 week.

### 2.4 Anti-CHI Effect of HGJD

#### 2.4.1 Animal Treatment

Thirty-six rats were randomly divided into six groups on average. Normal group (NG) and model group (MG) were received gavage of saline once daily for seven consecutive days. UDCA treated positive group (UDCA, 100 mg/kg), and three HGJD groups treated with low (LDG, 1.50 g/kg, extracts), medium (MDG, 3.01 g/kg, extracts), and high dosages (HDG, 6.02 g/kg, extracts) were received oral administration once daily for seven consecutive days. The adult dosage of HGJD is 50.50 g (crude drug) daily. Based on the body surface area index and lyophilized powder yield (33.12%) (Nair et al., 2018), the animal dosage of HGJD is calculated, and low animal dosage equals to the clinical dosage. On the fifth day, normal group was received the vehicle (soybean oil) treatment by gavage, and the other groups were received 60 mg/kg ANIT dissolved in an equal volume of soybean oil by gavage. According to previous studies, this dose was known to induce cholestasis.

The rats were maintained fasting for 12 h before receiving the last dose of HGJD. 1 h after the last dose, rats were anesthetized with pentobarbital. Blood samples were collected from abdominal aorta. After that, the rats were sacrificed. Liver samples were dissected, and rinsed once with ice-cold normal saline. One part of each liver sample was stored at −80°C for western blot and biochemical index analysis, and the remaining specimens were set in 10% PBS-buffered formalin for histopathological analysis.

#### 2.4.2 Assays of the Serum Enzymes and Components

The contents of alanine transaminase (ALT), aspartate transaminase (AST), alkaline phosphatase (ALP), γ-glutamyltranspeptidase (γ-GT), total bilirubin (TBIL), direct bilirubin (DBIL), and total bile acid (TBA) in serum were determined by commercial test kits.

#### 2.4.3 Histological Examination

The liver tissue fixed with 10% PBS-buffered formalin, were embedded in paraffin, serially sectioned (5 μm), and stained with nuclear dye (hematoxylin) and counterstain (eosin) for histological examination. Optical microscopy was applied to examine the histological section.

### 2.5 Identification of Serum Migrant Compounds of HGJD

#### 2.5.1 Animal Treatment

Six rats received oral administration of HGJD (6.02 g/kg) for 7 consecutive days. At fifth day, ANIT was orally administrated to rats in both the model group and the dose group. On the seventh day, after the orally administration of HGJD at 15 min, 30 min, 1, 2, 4, and 6 h, blood samples were taken from the angular vein of each rat. The serum samples collected from each rat at the different time points were mixed before sample processing.

#### 2.5.2 Preparation of Samples

10 ml of 50% methanol was applied to dissolve HGJD powder (0.1 g), and then the samples were extracted for 30 min by ultrasonic extraction. The mixture was centrifuged at 13,000 rpm maintained for 15 min. Filter membrane (0.22 μm) was used to filter the supernatant to obtain an injection sample.

1 ml serum sample was added with 3 ml methanol, then vortexed for 30 s and centrifuged at 13,000 rpm 4°C for 15 min to precipitate the protein. The supernatant was collected and dried with a nitrogen blowing concentrator. The residue was dissolved with 500 μl of 50% methanol, and repeated the centrifugation procedure. Then, a filter membrane (0.22 μm) was used to filter the supernatant to obtain an injection sample.

#### 2.5.3 UPLC-Q-Exactive MS/MS Analysis

The Vanquish UHPLC system (Thermo Fisher Scientific, United States) equipped with Q Exactive quadrupole-electrostatic field orbitrap high-resolution mass spectrometer was applied to analyze HGJD powder and serum samples. Samples were separated on an ACQUITY UPLC®BEH C_18_ column (2.1 × 100 mm 1.7 μm, Waters, United States). The mobile phase consisted of deionized water with 0.1% formic acid (A) and acetonitrile (B). The gradient elution procedure was as follows: 0–35 min, 5–95% B; 35–40 min, 95% B. The column temperature was maintained at 30°C. The flow rate was set at 0.4 ml/min.

All samples were analyzed in positive and negative ion modes, respectively. Mass spectrometry parameters were set as follows: nitrogen was selected as auxiliary gas and sheath gas. Flow rate was 10 L/min in positive mode and 35 L/min in negative mode. The voltage of the ion spray was 3 kV and −2.5 kV separately. The ion source temperature was kept at 320°C. Auxiliary gas heating temperature was maintained at 350°C. The fragment voltage was 50 V. Scan mass ratio was within the mass range of m/z 50–1000.

Compound Discoverer software (Thermo Fisher Scientific) was applied to analyze the LC-MS data. Components were identified by comparison of chemical information of reference substances, and initially identified with online databases (MZ cloud, MZ vault) and literatures.

### 2.6 Network Analysis

#### 2.6.1 Target Prediction of the Active Ingredients of HGJD Against CHI

The serum migrant components which were regarded as potential effective ingredients were applied to identify potential treatment targets of HGJD against CHI. Meanwhile, several online databases have contributed to the research of possible therapeutic targets. We first searched the CAS and 3D molecular structures of the serum migrant components on PubChem. Secondly, the CAS or 3D molecular structures were imported into TCMSP (http://tcmsp-e.com), Swiss Target Prediction (http://www.swisstargetprediction.ch/), and PharmMapper Server (Version 2017) (http://www.lilab-ecust.cn/pharmmapper/index.html) (Wang et al., 2017) to explore potential targets of the compounds. Then, “cholestasis”, “cholestatic hepatic injury” and “cholestatic liver injury” were used as keywords in the online database, including GeneCards (http://www.genecards.org/), Disgenet (https://www.disgenet.org/), OMIM (https://omim.org/), and TTD (Wang et al., 2020) (http://bidd.nus. edu. sg/group/ttd/) to screen for disease targets. Besides, standardization of all targets was conducted on the Uniprot database (https://www.uniprot.org/). Finally, intersection targets of CHI and HGJD were picked out by an online Venn analysis tool (http://bioinformatics.psb.ugent.be/webtools/Venn/).

#### 2.6.2 KEGG Pathways Analysis and GO Biological Process Enrichment

To clarify the function of the targets and their functions in signaling transduction, the DAVID 6.8 database was applied to evaluate the KEGG pathways and GO enrichment of the targets of HGJD against CHI.

#### 2.6.3 Network Construction

Three visualized networks were visualized by Cytoscape 3.7.1: 1) Drug-components-targets network was the interaction network among HGJD, potential active components of HGJD, and intersection targets; 2) Protein-Protein Interaction (PPI) network showed the importance of the targets of the potential active compounds of HGJD associated with CHI; 3) Components-Targets-Pathways network provided a systematic understanding of the complex network among drug, components, targets, pathways, and disease.

### 2.7 Metabolomics Analysis

#### 2.7.1 Sample Preparation

100 µl of thawed serum sample and 300 µl of methanol (precooled at −20°C) were added into 1.5 ml centrifuge tubes, and vortexed for 60 s, then solution was allowed to stand for 30 min at 4°C. Samples were centrifuged for 15 min at 12,000 rpm and 4°C to obtain supernatant. Each supernatant was added with internal standard (2-chlorophenoalanine 1 mg/ml), oscillated, mixed and filtered through 0.22 µm membrane to get the prepared sample for UPLC-MS analysis. Ultimately, 20 µl of each prepared sample was taken and mixed to obtain the QC sample (Dunn et al., 2011).

#### 2.7.2 Chromatography and Mass Spectrometry Conditions

Chromatographic separation was completed on Thermo Ultimate 3000 system which was equipped with a Hyper gold C_18_ (100 × 2.1 mm, 1.9 µm, and Thermo) column. Gradient elution of analyses was conducted with 0.1% formic acid in 5% acetonitrile(C) and 0.1% formic acid in acetonitrile (D) for positive ion mode. While, 0.05% acetic acid in 5% acetonitrile (A) and 0.05% acetic acid in acetonitrile (B) for negative mode. An incremental linear gradient of solvent B or D (v/v) was used as follows: 0–1.5 min, 0–20% B/D; 1.5–9.5 min, 20–100% B/D; 9.5–14.5 min, 100% B/D; 14.5–14.6 min, 100–5% B/D; 14.6–18 min, 5% B/D. The column flow rate and temperature were respectively set at 0.3 ml/min and 45°C. 3 μl of each sample was injected into UPLC-Q-Exactive MS/MS for analysis.

The experiments of ESI-MS^n^ were conducted on the Thermo Q Exactive mass spectrometer in positive and negative ion modes, respectively. Mass spectrometry parameters were set as follows: spray voltage was 3.0 kV in positive mode and −3.2 kV in negative mode. Sheath gas was kept at 45 arbitrary units, while auxiliary gas was 15 arbitrary units. The capillary temperature was maintained at 350°C. Full mass scan (m/z 70–1050) and HCD MS/MS spectra were recorded at a resolution of 70,000.

#### 2.7.3 Data Processing and Analysis

Raw data acquired by Proteowizard software (v3.0.8789) was transformed into mzXML format (Smith et al., 2006). Peaks recognition, filtration and comparison were executed by R (v3.3.2) XCMS package. Besides, peak intensity has been normalized, so as to facilitate comparison of different magnitudes of data. Then, Principal Component Analysis (PCA), Partial Least Squares Discriminant Analysis (PLS-DA), and Orthogonal-Partial Least Squares-Discriminant Analysis (OPLS-DA) methods were conducted by software SIMCA-P (v13.0) and R language ropes package to analyze data in different groups.

Components with marked changes in the groups (*p* < 0.05 and VIP >1) were chosen as differential biomarkers, which were tentatively identified with the exact molecular weight (error <20 ppm). Moreover, the MS/MS fragmentation patterns of compounds were matched by METLIN database (http://www.metlin.scipps.edu/) and MoNA database (https://mona.fiehnlab.ucdavis.edu//).

To reveal the changing trends of the potential biomarkers and the callback effect of HGJD, the heatmap analysis was carried out by MetaboAnalyst 5.0 (https://www.metaboanalyst.ca/MetaboAnalyst/) and R version 3.0.3. The pathways analysis of differential metabolites was conducted with MetaboAnalyst 5.0.

### 2.8 Regulation of PI3K/Akt/Nrf2 Pathway

#### 2.8.1 Western Blotting Assay

Samples have been processed and determined the concentrations of total protein with BCA kit, before western blotting assay to test the level of PI3K, p-Akt, GCLc, GCLm, and Nrf2. As for Nrf2, the nuclear and cytoplasmic extractions were carefully performed by extraction kits. Then, these extractions were detected by Western blot analysis for the presence of Nrf2. The samples with equal amount of total protein (100 μg) were electrophoresed on a twelve alkyl sulfate polyacrylamide gel system consisted of 5% stacking gel and 8–15% resolving gel. The electrophoresed products were transferred onto polyvinylidene fluoride membrane. The membranes were blocked in 5% skimmed milk powder for 0.5 h at room temperature, subsequently incubated with specific primary antibodies overnight, including PI3K (1:1000), p-Akt (1:1000), Nrf2 (1:1000), GCLc (1:1000), GCLm (1:1000), and β-actin (1:3000). Next, washed and incubated with appropriate secondary antibody for 30 min. Ultimately, membranes were developed with chemiluminescent detection reagents and visualized with film. The relative expressions of PI3K, p-Akt, Nrf2, GCLc, and GCLm were quantified through optical density value utilizing an image processing system.

#### 2.8.2 Biochemical Analysis of Antioxidant Compounds in Liver Tissue

Equal amount of Liver tissue (0.4 g) was carefully homogenized on ice with normal saline using a high speed tissue grinder to get a 1:10 (w/v) solution. Then the supernatants were tested using the GSH, MDA, and SOD assay kit according to the manufacturer’s instructions.

## 3 Results

### 3.1 Anti-CHI Effect Evaluation

**FIGURE 2:**
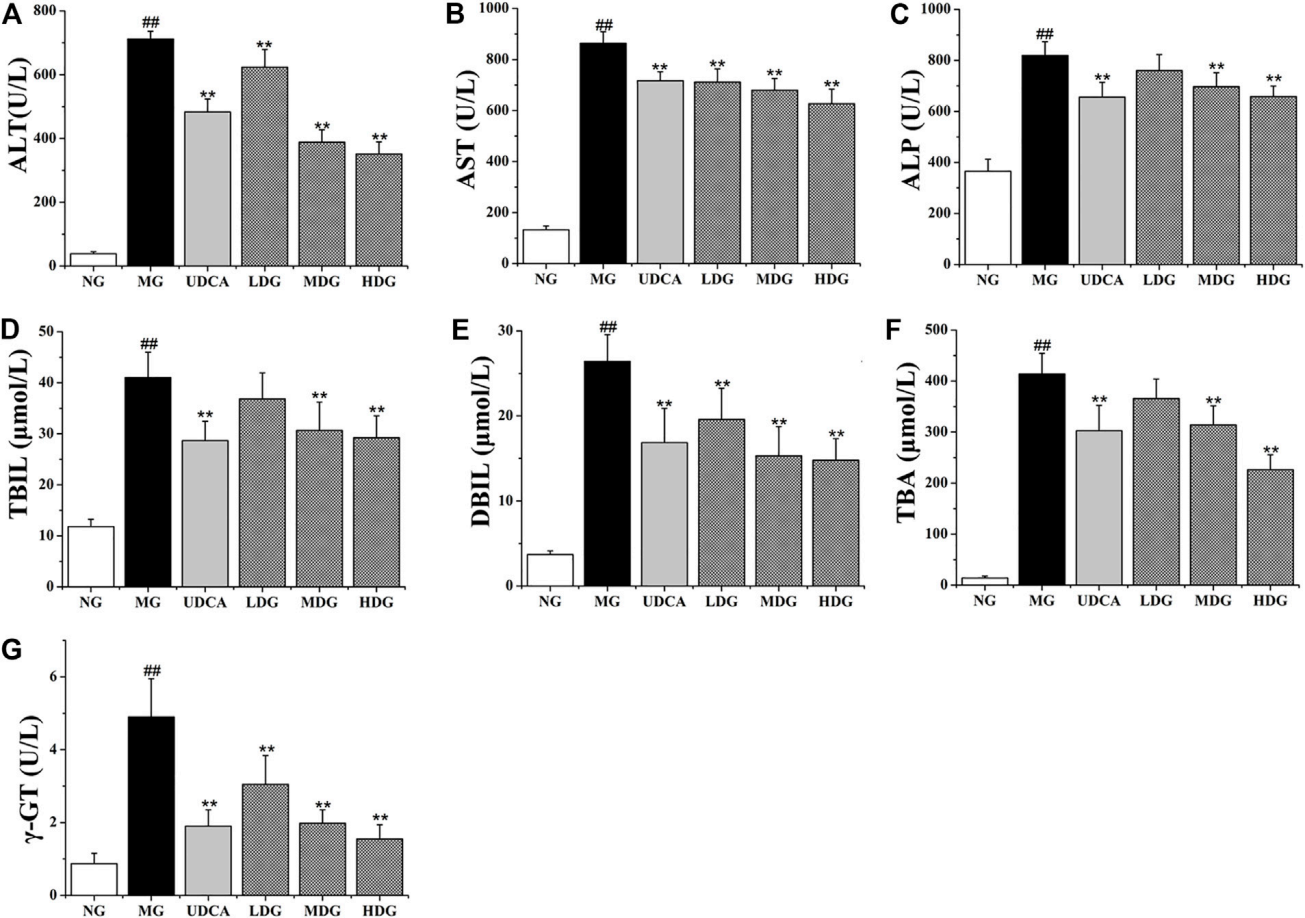
The effects of HGJD on serum biochemistry. The biomarkers of liver injury and biliary cell damage in the serum biochemistry were ALT **(A)**, AST **(B)**, ALP **(C)**, TBIL **(D)**, DBIL **(E)**, TBA **(F)** and γ-GT **(G)**. Data are expressed as the Mean ± SD (*n* = 6 in each group). ##*p* < 0.01 compared with the normal group; **p < 0.01 compared with the model group.

**FIGURE 3:**
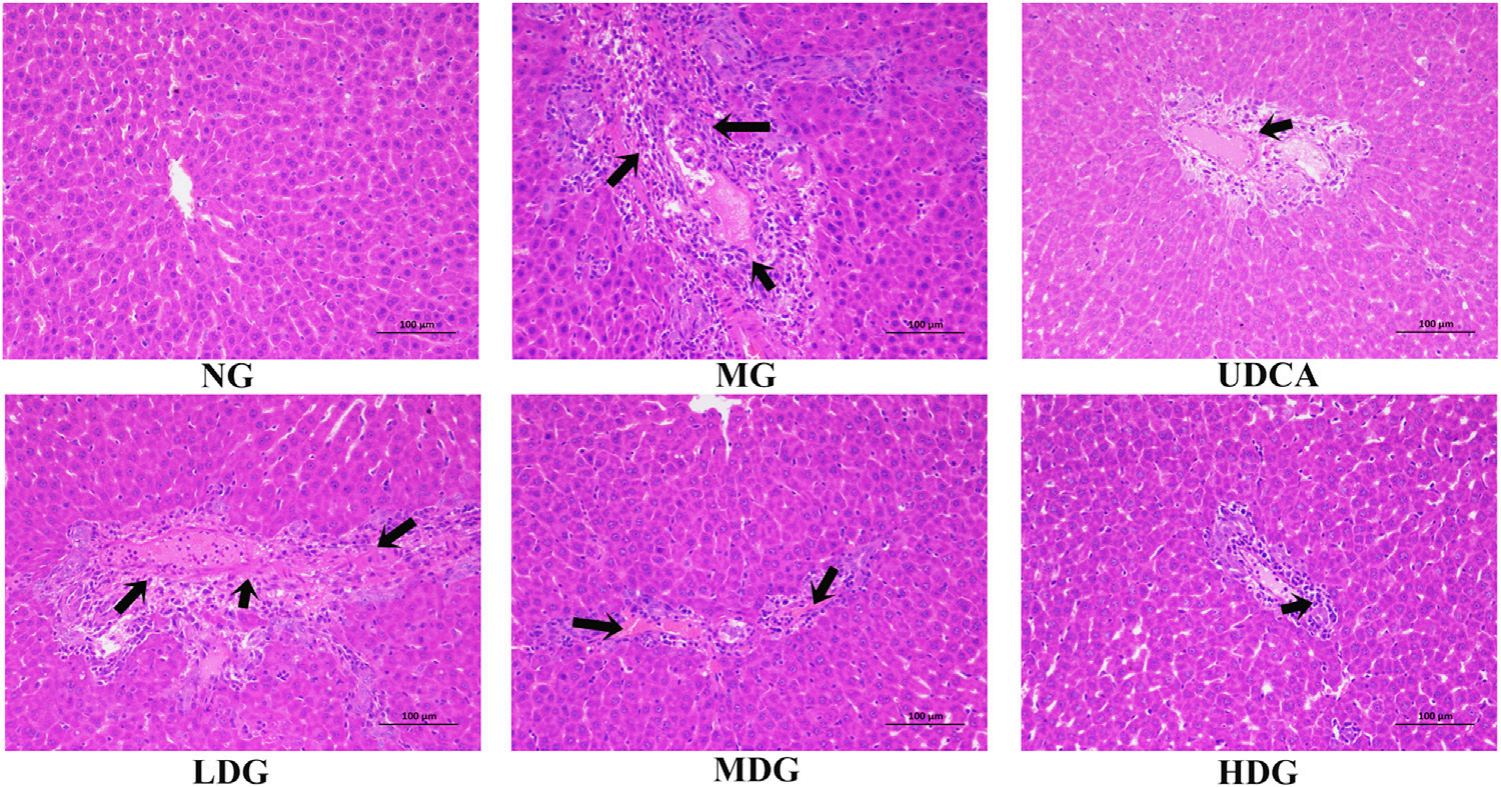
Representative histopathological section photos of liver specimens of rat with H&E staining (200 × magnification). Damage to hepatocytes is pointed by black arrows.

The levels of ALT, AST, ALP, TBIL, DBIL, TBA, and γ-GT in the model group were significantly higher than that in the normal group (*p* < 0.01), indicating that ANIT successfully induced the CHI in rats. The levels of all factors in HGJD treatment groups were dose dependently lower than those in model group (Figure 2). In addition, the high dose and medium dose of HGJD groups showed similar preventive effect with UDCA group on CHI (*p* < 0.01), while low dose showed weaker. As shown in Figure 3 **,** liver tissue of normal group displayed normal structure, while the specimens in the ANIT group observed spotty necrosis of liver cells. Administrating with 3.01 and 6.02 g/kg HGJD significantly reduced the trauma of liver tissue. The results showed that HGJD had a protective effect against ANIT-induced CHI.

### 3.2 Identification of Serum Migrant Compounds of HGJD

We established a UPLC-Q-Exactive MS/MS analysis method to analyze HGJD extracts and serum samples from rats after treatment of HGJD. The typical base peak chromatographs were shown in Supplementary Figure S1. On the basis of standards and related literatures, 31 compounds from HGJD in total were identified initially (Table 1 **)**. These compounds involved flavonoids, organic acids, phenols, alkaloids, and terpenoids. According to previous research, above compounds might originate from Citri Reticulatae Pericarpium Viride, Gardeniae Fructus, Paeoniae Radix Alba, Moutan Cortex, Alismatis Rhizaoma, Fritillariae Thunbergii Bulbus, and Citri Reticulatae Pericarpium in HGJD. Detailed source for each compound was shown in Table 1. In this study, prototype compounds absorbed into serum were mainly focused on. However, the metabolites in serum have not been identified and classified.

**TABLE 1:** Characterization of serum migrant compounds of HGJD by UPLC-Q-Exactive MS/MS.

NO.	Compound	RT (min)	Formula	Precursor ion	Predictived	Measured	Error (ppm)	MS2	Source	Reference
S1	Acetophenone	1.88	C_8_H_8_O	[M + H]^+^	121.0647	121.0652	4.13	103.0539, 91.0548, and 53.0391	CP	Zheng et al. (2020)
S2	Quinic acid	1.94	C_7_H_12_O_6_	[M−H]^−^	191.0561	191.0559	–1.05	173.0453, 85.0285	ZZ	Zhou et al. (2020)
S3	Nicotinamide	2.46	C_6_H_6_N_2_O	[M + H]^+^	123.0552	123.0554	1.63	88.0236, 80.0500, 56.9654, and 53.0392	CP	Zheng et al. (2020)
S4	Citric acid	3.23	C_6_H_8_O_7_	[M−H]^−^	191.0197	191.0195	–1.05	111.0080, 87.0078	BS	Xu et al. (2018)
S5	Geniposidic acid	4.67	C_16_H_22_O_10_	[M−H]^−^	373.114	373.1147	1.88	211.0611, 167.070, 123.0444, and 149.0602	ZZ	Wang et al. (2016)
S6	Shanzhiside	4.71	C_16_H_24_O_11_	[M−H]^−^	391.1245	391.1254	2.30	193.0507	ZZ	Zhou et al. (2020)
S7	Gardenoside	5.64	C_17_H_24_O_11_	[M−H]^−^	403.1245	403.1274	7.19	241.0719	ZZ	Fu et al. (2014)
S8	Jasminoside B	6.47	C_16_H_26_O_8_	[M + H]^+^	347.17	347.1700	0	185.1172, 167.1066	ZZ	Zhou et al. (2020)
S9	Methyl gallate	6.63	C_8_H_8_O_5_	[M−H]^−^	183.0298	183.0296	–1.09	168.0059, 124.0158	BS	Li et al. (2018)
									MDP	
S10	Oxypaeoniflorin	7.02	C_23_H_28_O_12_	[M−H]^−^	495.1508	495.1514	1.21	165.0555	BS	Zhan et al. (2018)
									MDP	
S11	Chlorogenic acid*	7.66	C_16_H_18_O_9_	[M + H]^+^	355.1023	355.1022	–0.28	—	ZZ	Wang et al., 2016; Zhou et al. (2020)
S12	Geniposide*	8.01	C_17_H_24_O_10_	[M + H]^+^	389.1442	389.1446	1.03	—	ZZ	Zhou et al. (2020)
S13	Peimisine	8.27	C_27_H_41_NO_3_	[M + H]^+^	428.3159	428.3157	–0.47	67.0549, 84.0813, 81.0704, and 79.0550	ZBM	Zhou et al. (2013)
S14	Peimine*	8.35	C_27_H_45_NO_3_	[M + H]^+^	432.3472	432.3470	–0.46	414.3004, 95.0860, and 67.0548	ZBM	Wang et al. (2014)
S15	Peiminine*	8.71	C_27_H_43_NO_3_	[M + H]^+^	430.3315	430.3313	–0.46	412.3204, 98.0967	ZBM	Zhou et al. (2013)
S16	Albiflorin*	8.78	C_23_H_28_O_11_	[M−H]^−^	525.1613	525.1619	1.28	357.1193, 121.0287	BS	Qi et al., 2014; Xiang et al. (2016)
S17	Paeoniflorin*	9.23	C_23_H_28_O_11_	[M−H]^−^	525.1613	525.1621	1.52	449.1466, 327.1079, 165.0550, and 121.0300	BS,MDP	Qi et al. (2014)
S18	Ebeiedinone	10.32	C_27_H_43_NO_2_	[M + H]^+^	414.3366	414.3364	–0.48	67.0548, 81.070, 91.0547, 93.0702, 105.0702, and 119.0857	ZBM	Zhou et al. (2013)
S19	Isoquercitrin	10.48	C_21_H_20_O_12_	[M + H]^+^	465.1027	465.1032	1.08	303.0497	CP,ZZ	Fu et al. (2014); Zhang et al. (2018)
S20	Puqiedinone	10.63	C_27_H_43_NO_2_	[M + H]^+^	414.3366	414.3359	–1.69	105.0702, 91.0547, 81.0704, 67.0548, and 55.0549	ZBM	Zhou et al. (2013)
S21	Narirutin*	10.90	C_27_H_32_O_14_	[M + H]^+^	581.1864	581.1855	–1.55	273.0750, 153.0181	CP,QP	Zheng et al. (2019)
S22	Hesperetin	11.43	C_16_H_14_O_6_	[M + H]^+^	303.0863	303.0859	–1.32	219.0640,171.0288,153.0181, 135.0439, 89.0390, and 67.0185	CP,QP	Zheng et al. (2020)
S23	Galloylpaeoniflorin	11.43	C_30_H_32_O_15_	[M + H]^+^	633.1814	633.1783	–4.90	153.0184	BS,MDP	(Xiang et al., 2016; Zhan et al., 2018)
S24	Hesperidin*	11.45	C_28_H_34_O_15_	[M−H]^−^	609.1824	609.1832	1.31	301.0719, 286.0480	CP,QP	(Xu et al., 2018; Zheng et al., 2019)
S25	Didymin*	13.90	C_28_H_34_O_14_	[M + H]^+^	595.2021	595.2021	0	287.0908	CP,QP	Tong et al. (2018)
S26	Naringenin	16.85	C_15_H_12_O_5_	[M−H]^-^	271.0611	271.0615	1.48	151.0041	CP,QP	Zheng et al. (2019)
S27	Paeonol*	19.10	C_9_H_10_O_3_	[M + H]^+^	167.0702	167.0701	–0.60	149.0596, 121.0648	BS,MDP	Xu et al. (2018)
S28	Nobiletin*	20.71	C_21_H_22_O_8_	[M + H]^+^	403.1387	403.1383	–0.99	403.1383, 388.1152, and 373.0914	CP,QP	Cai et al. (2019)
S29	Hepta-3*	21.72	C_22_H_24_O_9_	[M + H]^+^	433.1493	433.1489	–0.92	418.1256, 403.1018, and 385.0910	CP,QP	Cai et al. (2019)
S30	Tangeretin	22.45	C_20_H_20_O_7_	[M + H]^+^	373.1281	373.1276	–1.34	373.1280, 343.0809	CP,QP	Xu et al. (2018)
S31	Alisol B	25.97	C_30_H_48_O_4_	[M + H]^+^	473.3625	473.3626	0.21	437.342	ZX	Li et al. (2017)

CP, Citri Reticulatae Pericarpium (*Citrus reticulata* Blanco); QP, Citri Reticulatae Pericarpium Viride (*Citrus reticulata* Blanco); BS, Paeoniae Radix Alba (*Paeonia lactiflora* Pall); MDP, Moutan Cortex (*Paeonia suffruticosa* Andr.); ZZ, Gardeniae Fructus (*Gardenia jasminoides* Ellis); ZX, Alismatis Rhizaoma (*Alisma plantago-aquatica* Linn.); ZBM, Fritillariae Thunbergii Bulbus (*Fritillaria thunbergii* Miq.).

The substances with * in the table have been compared with the reference substance.

### 3.3 Network Analysis

#### 3.3.1 Targets Prediction of Potential Active Compounds of HGJD Against CHI

On the basis of 31 serum migrant compounds of HGJD, 596 targets of HGJD were picked. According to database analysis of disease targets, we obtained 962 targets. Subsequently, an online Venn analysis tool was applied to generate the Venn diagram. 159 common targets were regarded as potential targets of HGJD against CHI (Supplementary Figure S2, Table S2).

#### 3.3.2 KEGG Pathways Analysis and Gene Ontology (GO) Enrichment

**FIGURE 4:**
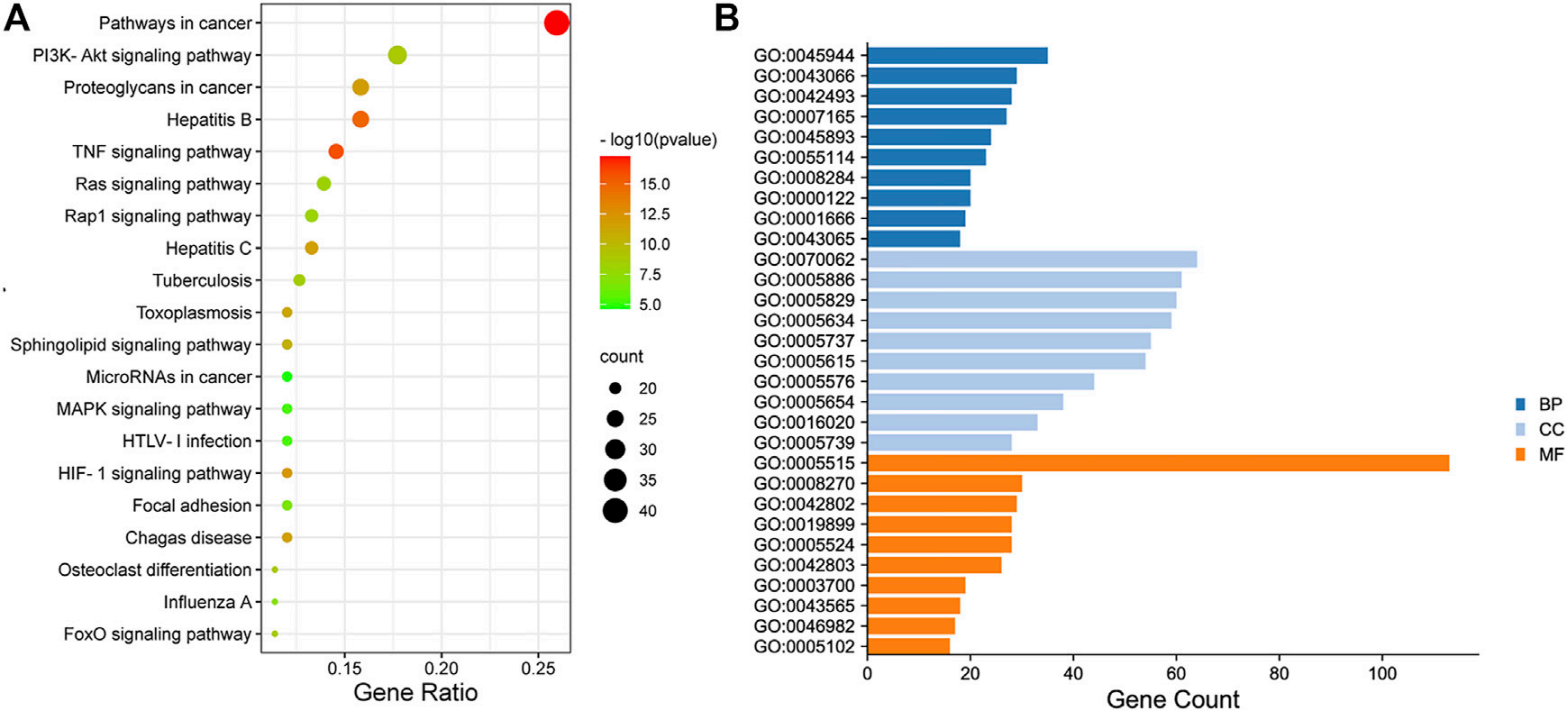
The Dotplot for KEGG pathway **(A)** and GO enrichment analysis of the predicted targets in Biological Process, Molecular Function, and Cell Composition **(B)**. In Figure 4A, the size of each node represents plentiful counts. Color indicates plentiful *p*-value. In Figure 4B, BP represents Biological Processes, CC represents Cellular Components, MF represents Molecular Functions.

The enrichment of signaling pathways was performed on DAVID database, and top 20 KEGG pathways which were selected according to the order of *p* value from small to large, were shown in Figure 4A; Supplementary Table S3. The results demonstrated that HGJD treating CHI mainly involved following pathways: pathways in cancer, PI3K-Akt signaling pathway and hepatitis B, *etc*.

The top10 results enriched by GO were displayed in Figure 4B; Supplementary Table S4. It was found that the target genes were involved in Cellular Components (CC), including extracellular exosome, plasma membrane, cytosol, and nucleus, *etc*. As for Biological Processes (BP), the key genes were mainly concentrated in negative regulation of apoptotic process, response to drug, signal transduction, and oxidation-reduction process. These functions closely correlated with protein binding, zinc ion binding, enzyme binding, and ATP binding in Molecular Functions (MF).

#### 3.3.3 Network Construction

Drug-components-targets network was conducted based on 31 serum migrant compounds of HGJD and 159 targets with therapeutic potential. (Supplementary Figure S3A; Supplementary Table S5). Besides, the protein-protein interaction network was established to screen the key targets of HGJD against CHI. As shown in Supplementary Figure S3B, the key targets involving in the anti-CHI effects of HGJD included MAPK1, AKT, MAPK3, and PIK3R1.

**FIGURE 5:**
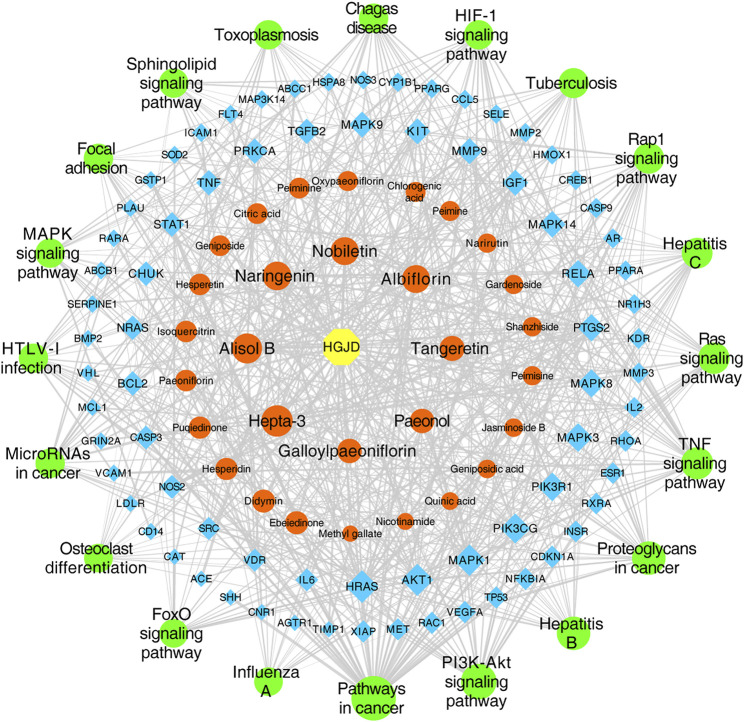
Network of drug components-targets-pathways. The yellow hexagon node represents drug, the orange round shape nodes represent absorbed ingredients in blood, the blue diamonds represent targets, the green round shape nodes represent pathways, and the edges represent the interactions between them. The size of nodes is proportional to their degree value.

The top 20 signaling pathways enriched by KEGG, targets involved in the top 20 signaling pathways, and the corresponding ingredients in HGJD were submitted to Cytoscape 3.7.1 to construction of the components-targets-pathways network (Figure 5). The network was consisted of 127 nodes (including 1 drug, 30 serum migrant compounds, 76 targets, and 20 pathways) and 728 edges. In addition, the network analysis result indicated that serum migrant compounds which had high degree value include hepta-3, alisol B, naringenin, nobiletin, albiflorin, tangeretin, paeonol, and galloylpaeoniflorin, might be the potential active ingredients of HGJD treating CHI.

### 3.4 Metabolomics

#### 3.4.1 Multivariable Data Analysis

**FIGURE 6:**
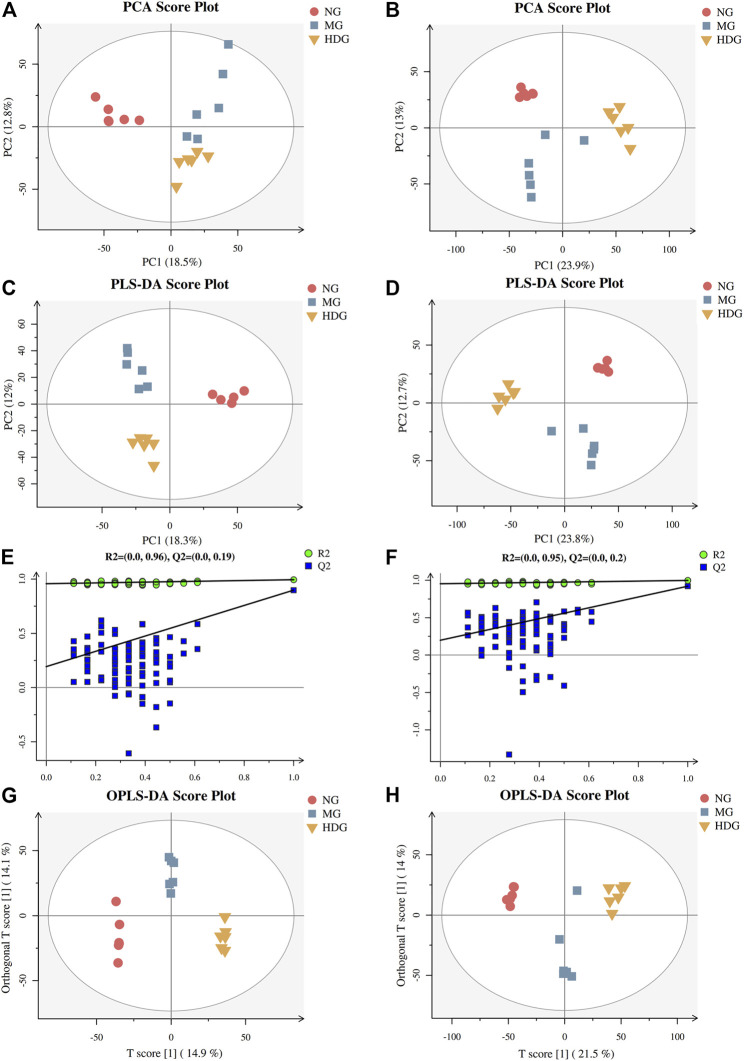
The results of multivariate statistical analysis. The PCA score graph in positive **(A)** and negative **(B)** ion mode. The PLS-DA score graph in positive **(C)** and negative **(D)** ion mode. Permutations graph in positive **(E)** and negative **(F)** ion mode. The OPLS-DA score graph in positive **(G)** and negative **(H)** ion mode.

The typically based peak intensity chromatograms of serum samples in different groups were shown in Supplementary Figure S4, S5. Meanwhile, QC samples could cluster together, indicating that the instrument was stable (Supplementary Figure S6). The PCA and PLS-DA score plots (ESI+: R2X = 0.435, R2Y = 0.994, and Q2 = 0.898; ESI-: R2X = 0.480, R2Y = 0.997, and Q2 = 0.92) indicated that the normal group, model group and high dose of HGJD treatment group were separated from each other (Figures 6A–D), the combination of histological and biochemical examination confirmed the reliability of the CHI model and reflected the regulatory effect of HGJD on metabolites. Simultaneously, permutation test indicated that PLS-DA model was not over-fitted. **(** Figures 6E,F **)**. The complexity of the model was reduced using OPLS-DA and the interpretation of the model was improved (Figures 6G,H). The results showed that the metabolites in each group have achieved complete separation (ESI+: R2X = 0.372, R2Y = 0.999, Q2 = 0.859; ESI-: R2X = 0.356, R2Y = 0.985, and Q2 = 0.901) in two ion modes. Further, VIP value in OPLS-DA was one of the important factors for identifying differential metabolites.

#### 3.4.2 Identification of Differential Biomarkers

**FIGURE 7:**
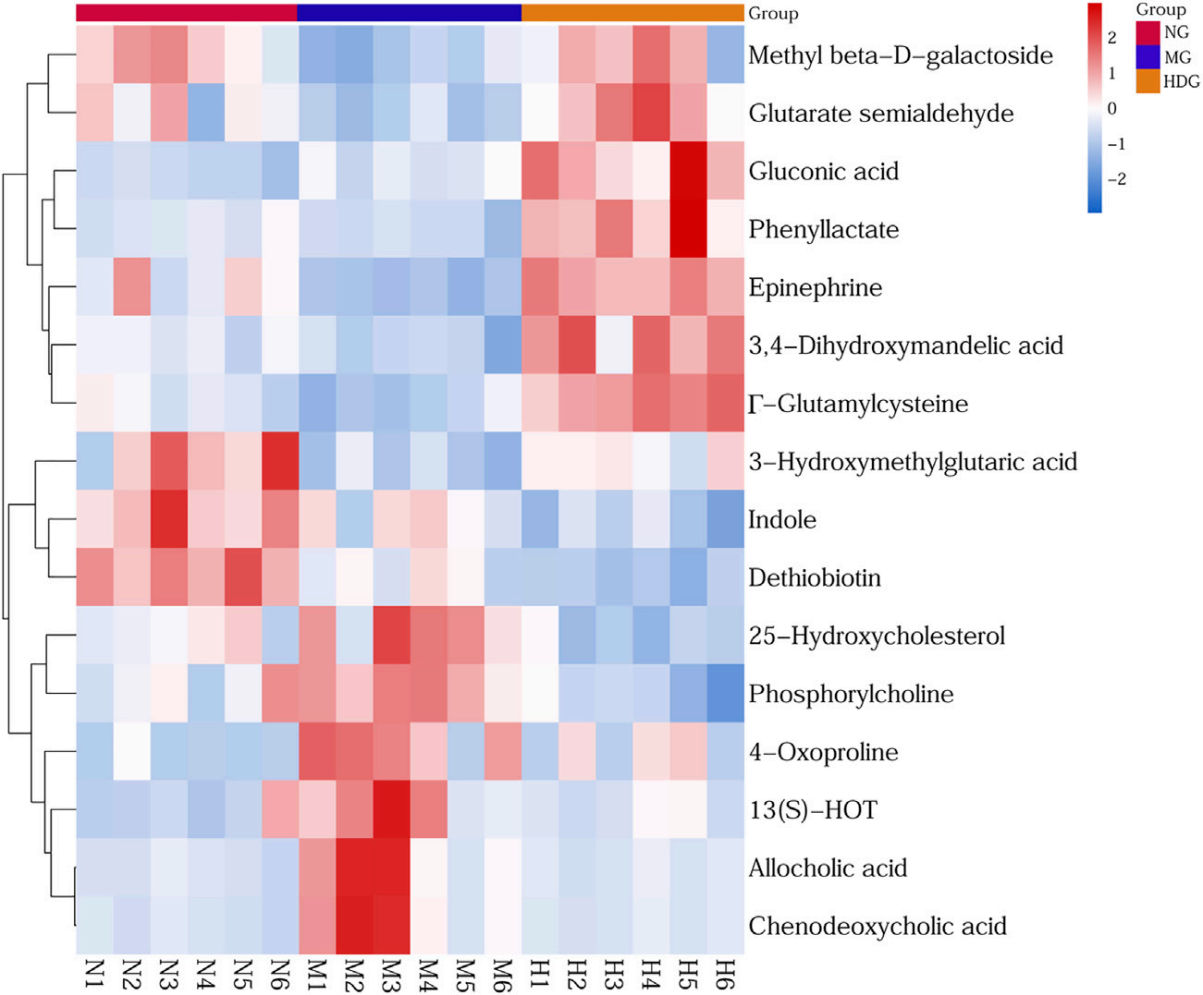
Heatmap of differential metabolites. Color changed from blue to red, corresponding to a progressive increase in concentration.

OPLS-DA models and *t*-test were applied to distinguish the differential metabolites induced by ANIT and improved by HGJD. Differential biomarkers were screened by a criterion of *p*-value < 0.05 and VIP >1. Based on literature reports and four online databases including Human Metabolome Database (HMDB) (http://www.hmdb.ca), Metlin (http://metlin.scripps.edu), massbank (http://www.massbank.jp/), LipidMaps (http://www.lipidmaps.org), and mzclound (https://www.mzcloud.org), the differential biomarkers were tentatively identified. In total, 16 differential metabolites were screened out. As shown in Table 2 **,** 7 metabolites were up-regulated and 9 metabolites were down-regulated with ANIT intervention in model group. Compared to this trend, the level of 13 metabolites was significantly reversed (*p* < 0.05) in the high dose of HGJD treatment group. The heatmap of 16 biomarkers was displayed in Figure 7, which demonstrated that ANIT treatment could influence the level of endogenous metabolites notably, while HGJD treatment had beneficial effects for the recovery of metabolite disorders.

**TABLE 2:** List and change trends of differential metabolites in the rats serum induced by ANIT.

No	Name	RT(min)	Formula	Exact mass	Error (ppm)	KEGG	Type	Trend	
								MG *vs* NG	HDG *vs* MG
1	Methyl beta-D-galactoside	0.69	C_7_H_14_O_6_	194.0790	6.69	C03619	[M + H]^+^	↓	↑
2	Epinephrine	1.03	C_9_H_13_NO_3_	183.0895	12.25	C00788	[M + H]^+^	↓	↑
3	3-Hydroxymethylglutaric acid	1.10	C_6_H_10_O_5_	162.0528	8.96	C03761	[M−H]^−^	↓	↑
4	Gluconic acid	1.27	C_6_H_12_O_7_	196.0583	2.79	C00257	[M−H]−	↑	↑
5	Glutarate semialdehyde	2.81	C_5_H_8_O_3_	116.0473	14.90	C03273	[M−H]−	↓	↑
6	3,4-Dihydroxymandelic acid	2.97	C_8_H_8_O_5_	184.0370	3.54	C05580	[M−H]−	↓	↑
7	Indole	3.02	C_8_H_7_N	117.0578	4.05	C00463	[M + H]^+^	↓	↓
8	Phenyllactate	4.18	C_9_H_10_O_3_	166.0630	5.06	C05607	[M−H]−	↓	↑
9	25-Hydroxycholesterol	5.62	C_27_H_46_O_2_	402.3498	2.56	C15519	[M−H]−	↑	↓
10	Allocholic acid	6.14	C_24_H_40_O_5_	408.2876	7.46	C00695	[M + H]^+^	↑	↓
11	Chenodeoxycholic acid	6.14	C_24_H_40_O_4_	392.2927	15.10	C02528	[M + H]^+^	↑	↓
12	Dethiobiotin	6.54	C_10_H_18_N_2_O_3_	214.1317	11.18	C01909	[M−H]−	↓	↓
13	Phosphorylcholine	7.27	C_5_H_15_NO_4_P	184.0739	1.02	C00588	[M + H]^+^	↑	↓
14	4-Oxoproline	8.57	C_5_H_7_NO_3_	129.0426	1.54	C01877	[M + H]^+^	↑	↓
15	13(S)-HOT	8.65	C_18_H_30_O_3_	294.2195	1.04	C16316	[M + H]^+^	↑	↓
16	Γ-Glutamylcysteine	9.63	C_8_H_14_N_2_O_5_S	250.0623	0.97	C00669	[M−H]−	↓	↑

↑: Compound is up-regulated. ↓: Compound is down-regulated.

#### 3.4.3 Metabolic Pathway Analysis

**FIGURE 8:**
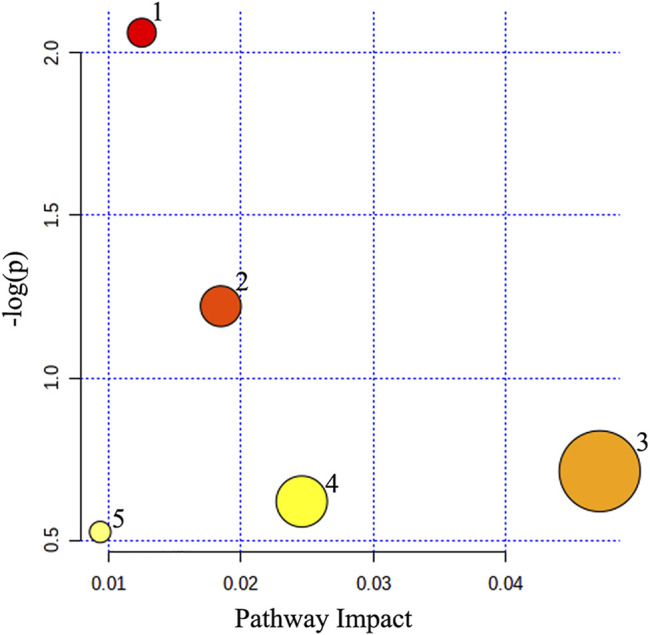
Pathway analyses of differential metabolites in serum sample.

Metabolic pathway analysis was carried out to explore the underlying molecular functions of these serum differential metabolites. The result revealed that 5 pathways were affected by the administration of HGJD, including primary bile acid biosynthesis, tyrosine metabolism, pentose phosphate pathway, glutathione metabolism and glycerophospholipid metabolism (Figure 8; Table 3).

**TABLE 3:** Results of metabolic pathway analysis.

No	Pathway name	Total	Hits	*p*-value	–log(*p*)	Impact
1	Primary bile acid biosynthesis	46	3	0.008671	2.0619	0.0125
2	Tyrosine metabolism	42	2	0.060225	1.2202	0.0185
3	Pentose phosphate pathway	22	1	0.193790	0.7127	0.0471
4	Glutathione metabolism	28	1	0.240200	0.6194	0.0246
5	Glycerophospholipid metabolism	36	1	0.298210	0.5255	0.0094

#### 3.4.4 Signaling Networks

**FIGURE 9:**
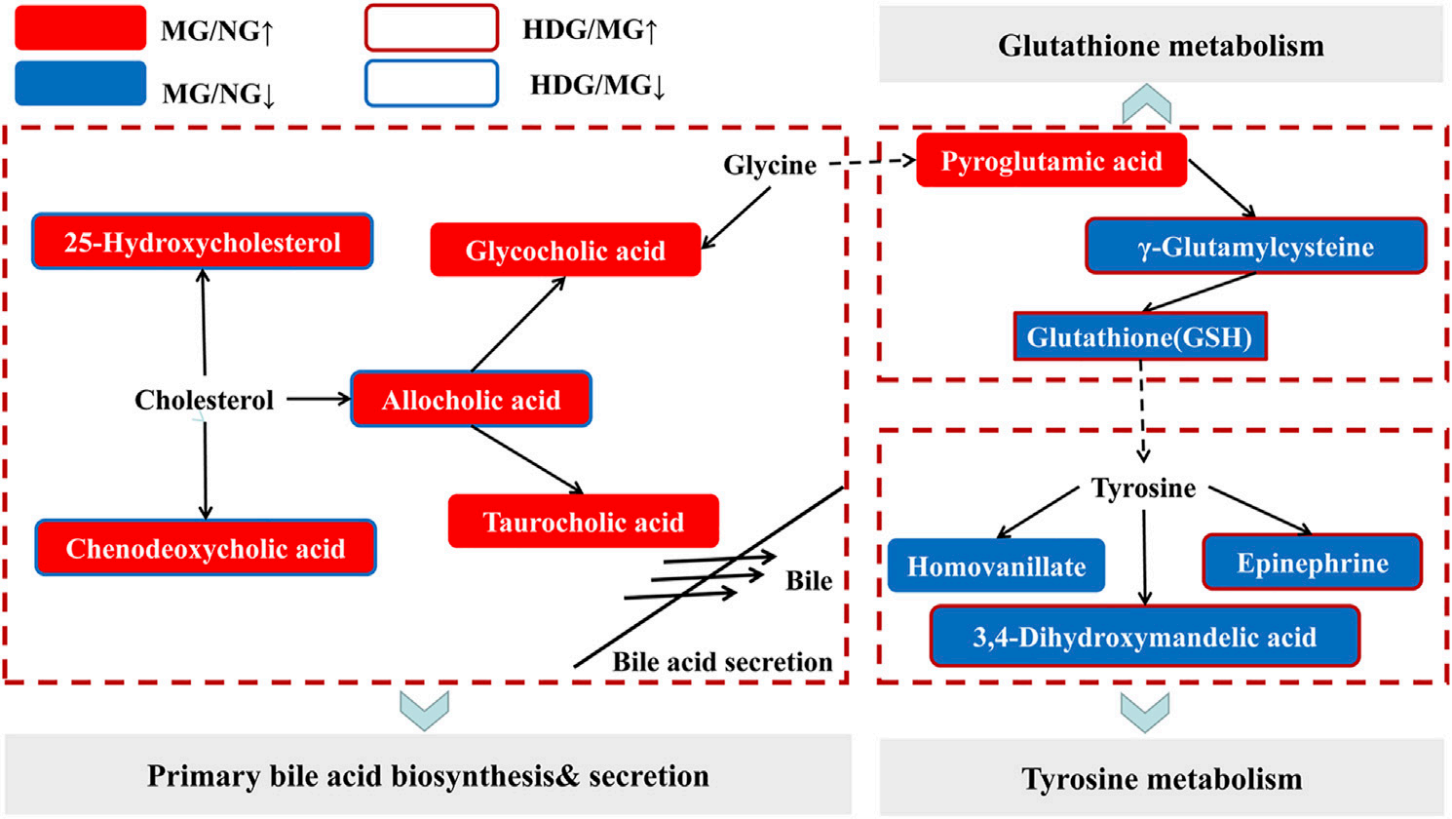
Sketch map of the metabolic pathway correlated with CHI and HGJD therapy. The red and blue solid boxes represent that metabolites were significantly elevated or reduced in the model group when contrasted with normal group, respectively. The boxes with red and blue border indicated that metabolites were significantly increased or decreased in the HGJD group when compared with model group, respectively.

We imported the identified differential metabolites into the KEGG (http://www.kegg.jp/) to find signaling pathways correlated with them. Moreover, relationships among different signaling pathways were analyzed based on literatures. As shown in Figure 9, primary bile acid biosynthesis, tyrosine metabolism and glutathione metabolism might be related to the anti-CHI effect of HGJD. Meanwhile, when compared with model group, the levels of 25-Hydroxycholesterol, cholic acid, and chenodeoxycholic acid were significantly down-regulated by HGJD. Conversely, the levels of Γ-Glutamylcysteine, Epinephrine and 3, 4-Dihydroxymandelic acid were significantly increased by HGJD.

#### 3.5 Regulation of PI3K/Akt/Nrf2 Pathway

##### 3.5.1 Western Blotting Assay

**FIGURE 10:**
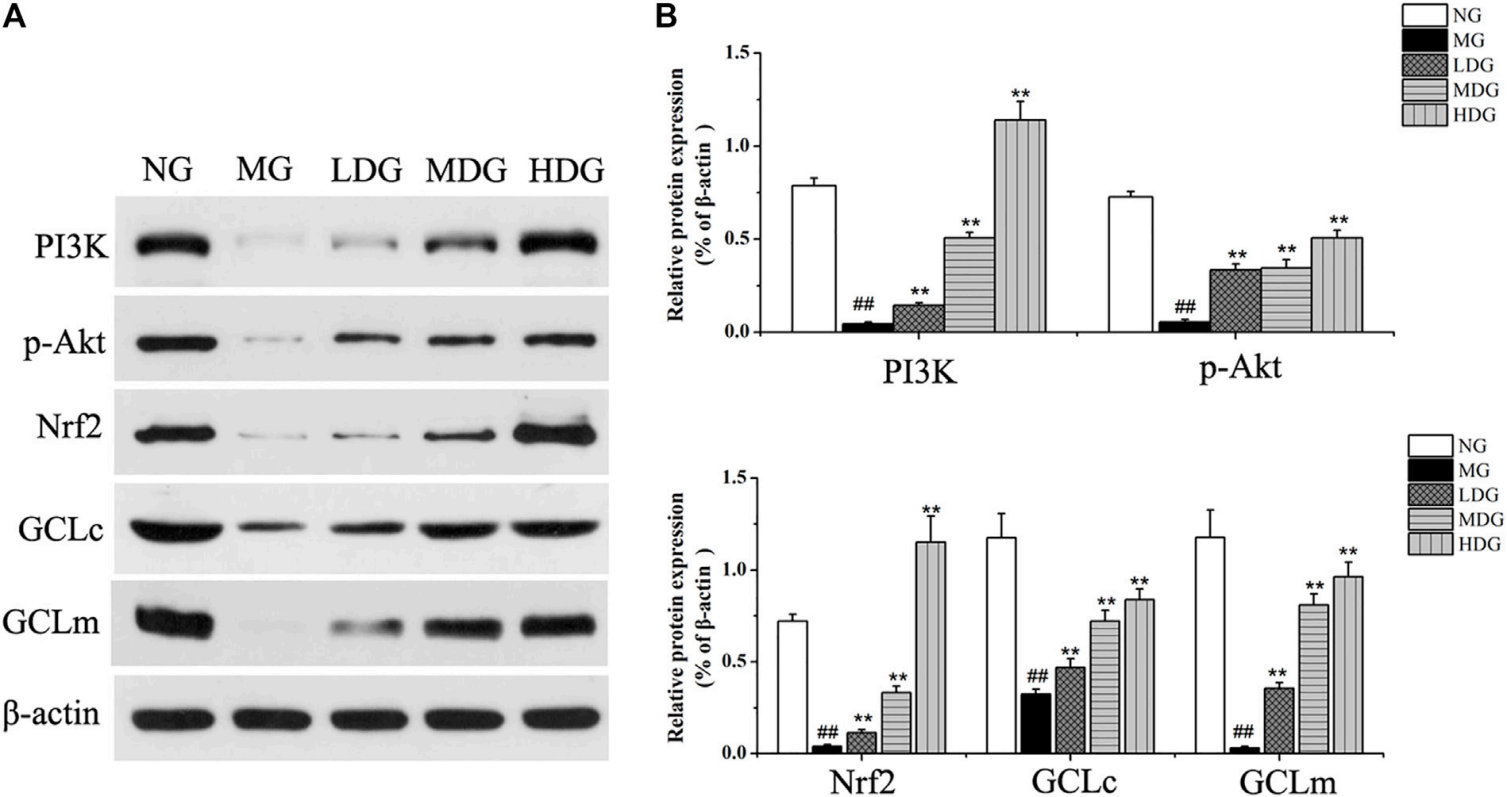
The effects of HGJD on the expression of PI3K/Akt/Nrf2 signaling pathway. The effect of HGJD pretreatment on PI3K p-Akt, Nrf2, GCLc, and GCLm protein expression after ANIT treatment **(A)** and the optical density analysis of the protein bands intensity **(B)**. Data are expressed as the mean ± SD, ##*p* < 0.01 contrasted with the normal group. **p < 0.01 contrasted with the model group.

Integrating liver biochemistry, network analysis and metabolomics results, PI3K/Akt pathway, primary bile acid biosynthesis as well as secretion, and glutathione metabolism might be involved in the anti-CHI effect of HGJD. Meanwhile, accumulated evidence has shown that activation of PI3K/Akt pathway could induce nuclear translocation of Nrf2, and then affect GSH synthesis, which was an important mechanism of nature product protection against hepatic oxidative injury (Ma et al., 2015; Jin et al., 2019; Mukherjee et al., 2021). Therefore, western blotting was used to test and verify the effect of HGJD on PI3K/Akt/Nrf2 signaling pathway. As displayed in Figure 10, the protein expressions of PI3K, p-Akt, Nrf2, GCLc, and GCLm were decreased in ANIT induced model group. Whereas, these reduced protein levels were significantly increased after the treatment with HGJD.

#### 3.5.2 Biochemical Analysis of Antioxidant Compounds in Liver Tissue

**FIGURE 11:**
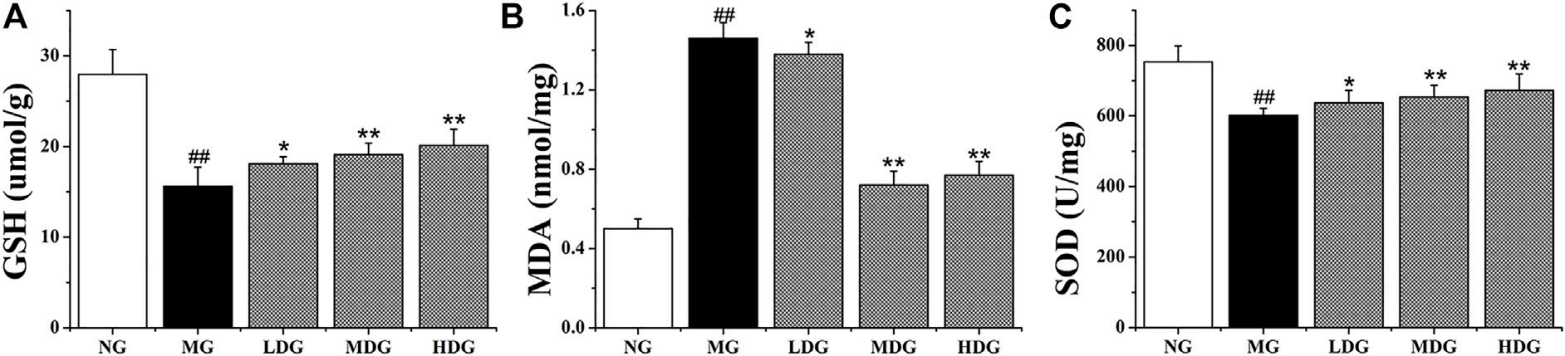
Effects of HGJD on GSH **(A)**, MDA **(B)**, and SOD **(C)** in liver specimens in ANIT-induced CHI rats. Data are expressed as the Mean ± SD (*n* = 6 in each group). ##*p* < 0.01 contrasted with the normal group; *p < 0.05 compared with the model group; **p < 0.01 contrasted with the model group.

As reported in literatures, activation of PI3K/Akt/Nrf2 pathway could promote the imbalance between antioxidant system and oxidative system, which was closely related to mitigation of oxidative stress-induced injuries (Shi et al., 2018). Antioxidant compounds content (GSH, SOD) which could scavenge ROS, were significantly reduced with treatment of ANIT (*p* < 0.01). In UDCA group, medium dose and high dose of HGJD group, the levels of these compounds in liver were significantly reversed, while the antioxidant effect of low dose of HGJD was weak (Figures 11A,C). The hepatic MDA which was one of oxidative stress products was significantly increased in the model group (*p* < 0.01). The treatment with different dose of HGJD significantly reversed the increase of MDA induced by ANIT (Figure 11B). These results demonstrated that the potential mechanism of HGJD to alleviate CHI might correlate with the activation of PI3K/Akt/Nrf2 pathway to induce antioxidant synthesis.

## 4 Discussion

In this study, HGJD significantly decreased the biochemical indicators which were related to CHI, and improved the inflammatory infiltration induced by ANIT. The results suggested that HGJD had a protective effect against ANIT-induced CHI. In terms of biochemical results, the effect of HGJD was not as good as UDCA, which might be related to overall regulation, slowness, and long-lasting characteristics of TCM treatment. In addition, serum pharmacochemistry combined with network analysis was applied to analyze drug-target interactions and identify potentially active components of HGJD. Moreover, mechanisms of anti-CHI effect of HGJD were explored by integrating network analysis and metabolomics, and the predicted main pathway was validated in the level of molecular biology. Although dosage in our study is within the range stipulated by Chinese Pharmacopoeia (2020 Edition), it is still lager than the dosage recommends for animal studies (Heinrich et al., 2020). Further pharmacological and toxicological studies are needed to guide the determination of clinical dose range.

Through literature research, we found that metabolic enzyme activity and gut microbiota might change under disease conditions, which could affect the absorption of pharmaceutical ingredients (Feng et al., 2020; Kummen and Hov, 2019; Pang et al., 2017; Qi et al., 2020). Therefore, in this experiment, the ANIT-induced CHI rats were selected to study serum pharmacochemistry, so as to better reflect absorption of HGJD components under the pathological state of CHI. Moreover, the network analysis was used to screen for potential active components of HGJD. The present results showed that hepta-3, alisol B, naringenin, nobiletin, albiflorin, tangeretin, paeonol, and galloylpaeoniflorin were the pivotal compounds of the compound-target network of HGJD against CHI. As reported in literature, hepta-3, naringenin, tangeretin, and nobiletin could reduce oxidative stress-induced liver injury by Nrf2 related pathway (Lin et al., 2019). Due to the regulation of transporters and enzymes mediated by FXR, Alisol B 23-acetate has shown the anti-CHI effect in animal experiment (Meng et al., 2015). Albiflorin was the one of the main active compounds of *Paeonia lactiflora* Pall against CHI (Jiang et al., 2012). Paeonol could alleviate live injury via anti-inflammatory, anti-oxidative and anti-apoptosis activity (Ding et al., 2016; Gong et al., 2017). These active components and their mixtures might play a vital role in protecting effect of HGJD against ANIT-induced CHI. However, the relationships among those compounds were complex, which needed to be explored in future work.

Past work has shown that inhibition of PI3K/Akt pathway might aggravate liver damage (Han et al., 2010; Liu W et al., 2019). Besides, natural products have been demonstrated to have hepatoprotective activity via up-regulated the expression of Nrf2 by activating the PI3K/Akt pathway (Zhang et al., 2017). From the results of network analysis, PI3K/Akt signaling pathway had a significant correlation with the key targets identified in our study. Furthermore, as reported in literature, under acute and chronic liver disease conditions, Nrf2 was activated and relieved oxidative stress-induced injuries by regulating genes expression of cytoprotective enzymes, which promoted GSH synthesis, and inhibited ROS generation (Bataille and Manautou, 2012; Xu et al., 2019). Based on our serum metabolomics research, glutathione metabolism might contribute to the anti-CHI effect of HGJD. Thus integrating results from network analysis and metabolomics showed that PI3K/Akt/Nrf2 signaling pathway related to glutathione (GSH) synthesis might be one of the major pathways interrelated to anti-CHI function of HGJD. Therefore, we further studied the connection among GSH synthesis, Nrf2 expression, and PI3K/Akt signaling pathways. As demonstrated in this study, HGJD could improve the protein expressions of PI3K, p-Akt, Nrf2, GCLc and GCLm, and increase GSH and SOD content. The results effectively corroborated the above prediction results, suggested that this integrated strategy was feasible.

As for CHI, excessive accumulation of bile acids in liver and systemic circulation could induce live cell damage (Gonzalez-Sanchez et al., 2016), which was related to bile acid-induced initiation of apoptosis and oxidative stress increase in liver cells (Sokolovic et al., 2013). The non-targeted metabolomics in our research showed that the therapeutic effect of HGJD was correlated with primary bile acid biosynthesis as well as secretion, and glutathione metabolism. Moreover, as reported in literature, farnesoid X receptor (FXR) played an important role in regulating bile acid. Meanwhile, oxidative stress in FXR-null mice increased spontaneously, which might be attributed to the continuous increase in hepatic bile acids level (Nomoto et al., 2009). However, there was a complex network of biological signaling pathways, and might be a clear tissue-specific role of FXR in the liver and intestine (Cheng, et al., 2021; Stofan and Guo, 2020). FXR and the molecular mechanism by which it regulates bile acid and oxidative stress are valuable for further research.

In conclusion, this study showed that HGJD could alleviate CHI. A comprehensive method based on network analysis, metabolomics and *in vivo* validation experiment was used to investigate the mechanism of HGJD in treatment of CHI. PI3K/Akt/Nrf2 signaling pathway related to GSH synthesis has been identified as the staple pathway correlated with the effects of HGJD against CHI. Totally, 31 compounds originated from HGJD have been identified in the serum sample. The pivotal compounds in the network analysis were predicted as potential active components. In the future, other signaling pathways predicted in our study should be investigate, and the potential active ingredients can be used to improve the quality control of HGJD.

## Data Availability

The original contribution presented in the study are included in the article/Supplementary Material; further inquiries can be directed to the corresponding authors.
