# Supplementary material for: Integrating Network Analysis and Metabolomics to Reveal Mechanism of Huaganjian Decoction in Treatment of Cholestatic Hepatic Injury
Source: Front Pharmacol. 2022 Jan 19;12:773957. doi: 10.3389/fphar.2021.773957 (PMC8807561; doi:10.3389/fphar.2021.773957)
Supplement: Supplementary file 1 [file DataSheet1.doc]

***Supplementary Material***

# Supplementary Figures and Tables

1.1 Supplementary Figures


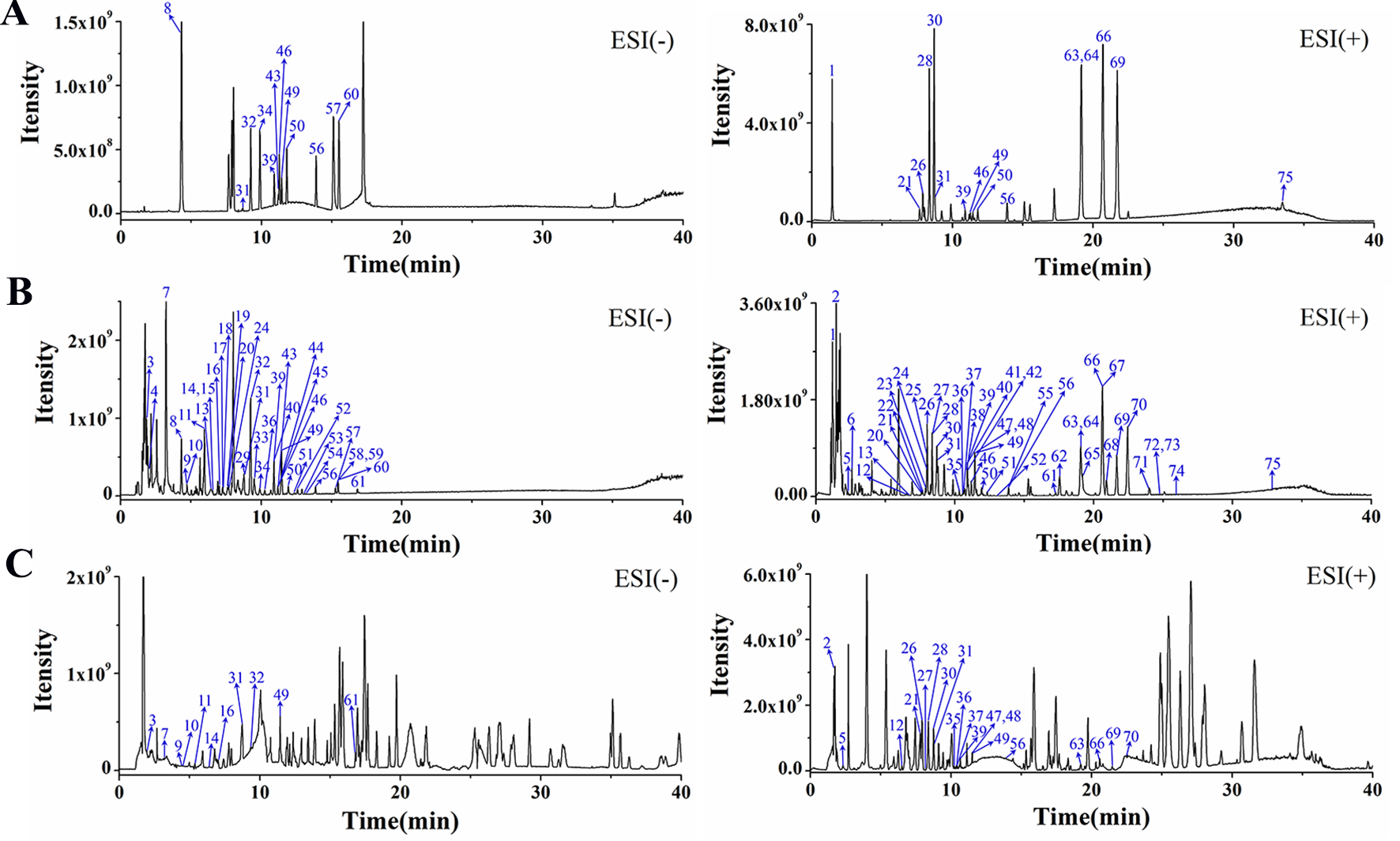


**Figure S1.** Base Peak chromatographs of 22 mixed standards (A), HGJD lyophilized sample (B), serum sample collected from model rats after oral administration of HGJD (C) based on UPLC-Q-Exactive MS/MS in negative ion mode (left side) and positive ions mode (right side). The relationship of serial number in serum sample (S1-S31) and HGJD sample (1-75) was shown in Table S1.


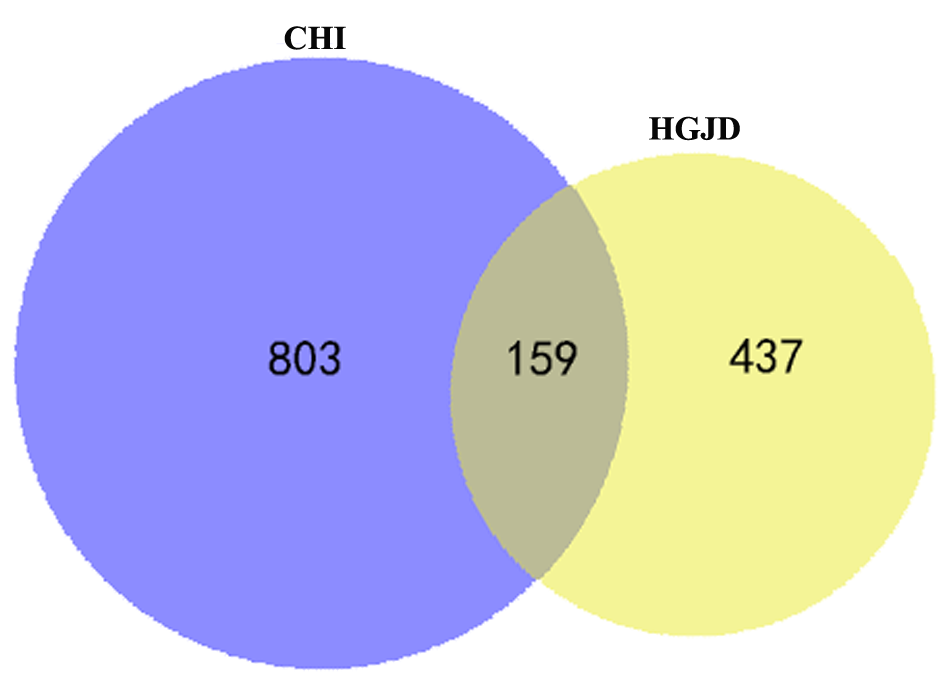


**Figure S2** Venn diagram of the common targets of HGJD and CHI


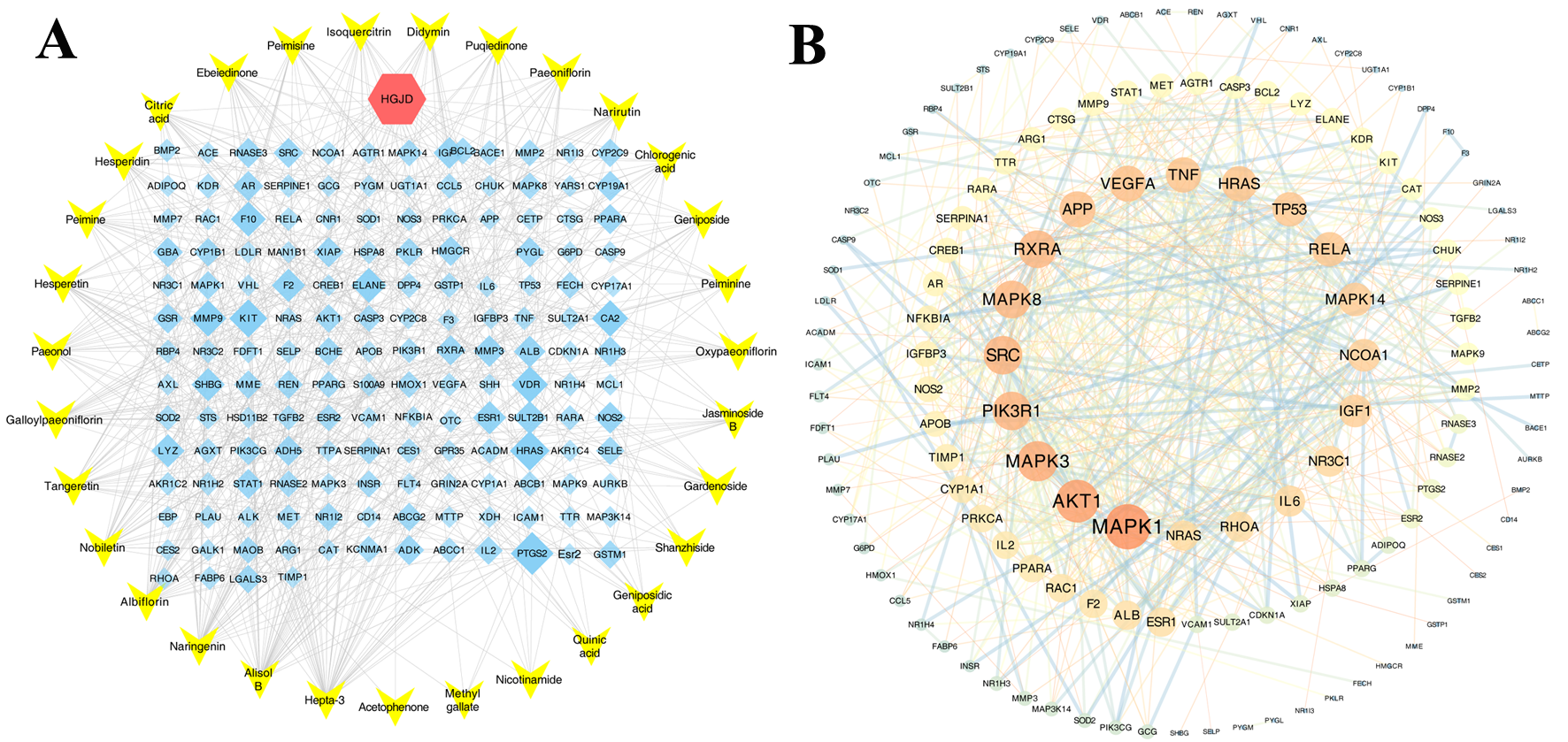


**Figure S3** Drug-compound-target network (**A**) and PPI network of potential targets for HGJD treatment of CHI (**B**). The red hexagon represents drug, the yellow "V" arrows represent the serum migrant compounds of HGJD, and the blue diamonds represent targets. The node size changed from small to large, and the color changed from blue to orange, indicating that the degree of target from small to large in the PPI network diagram.


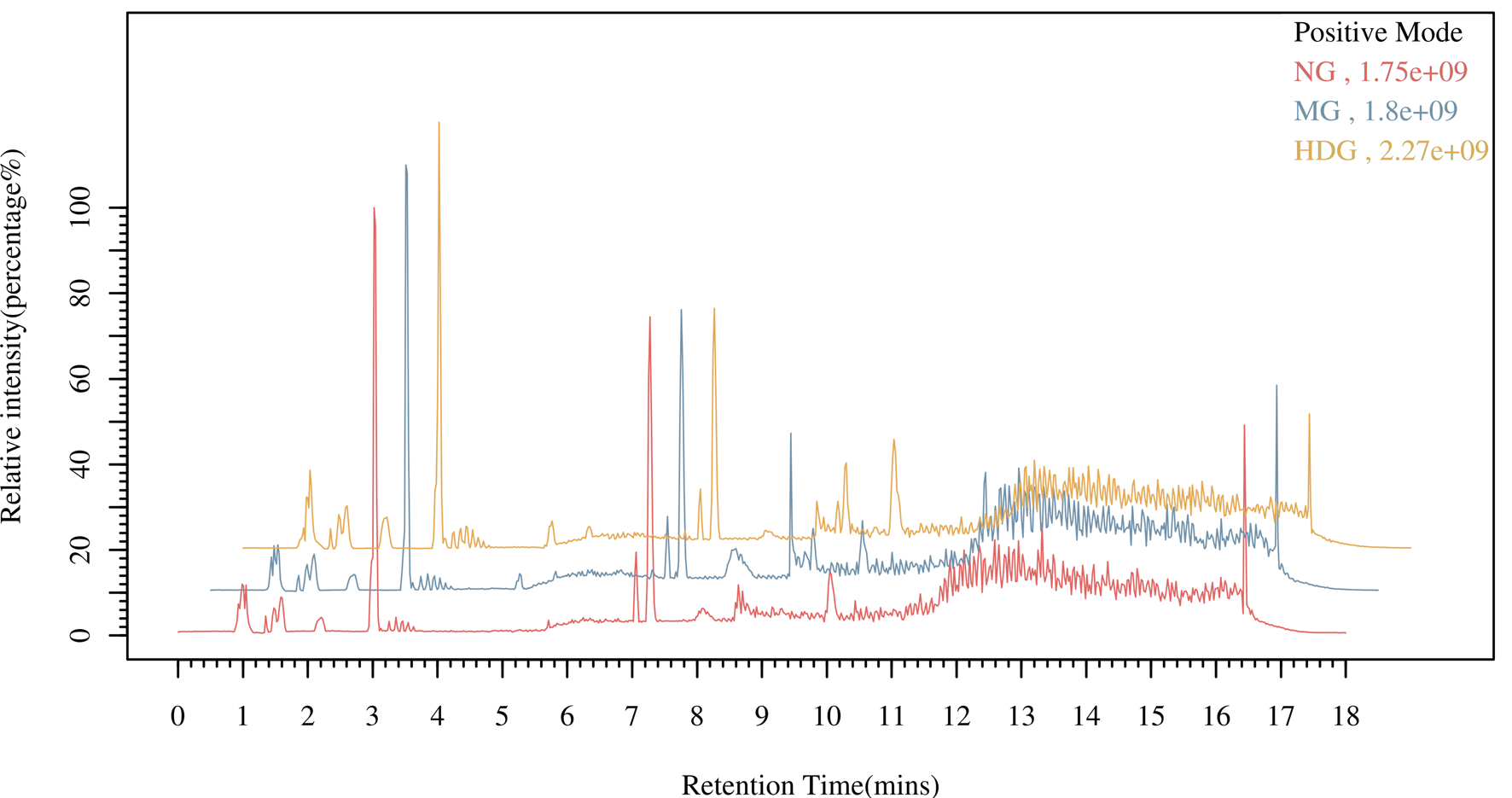


**Figure S4** Base peak chromatograms of typical serum sample in positive mode.


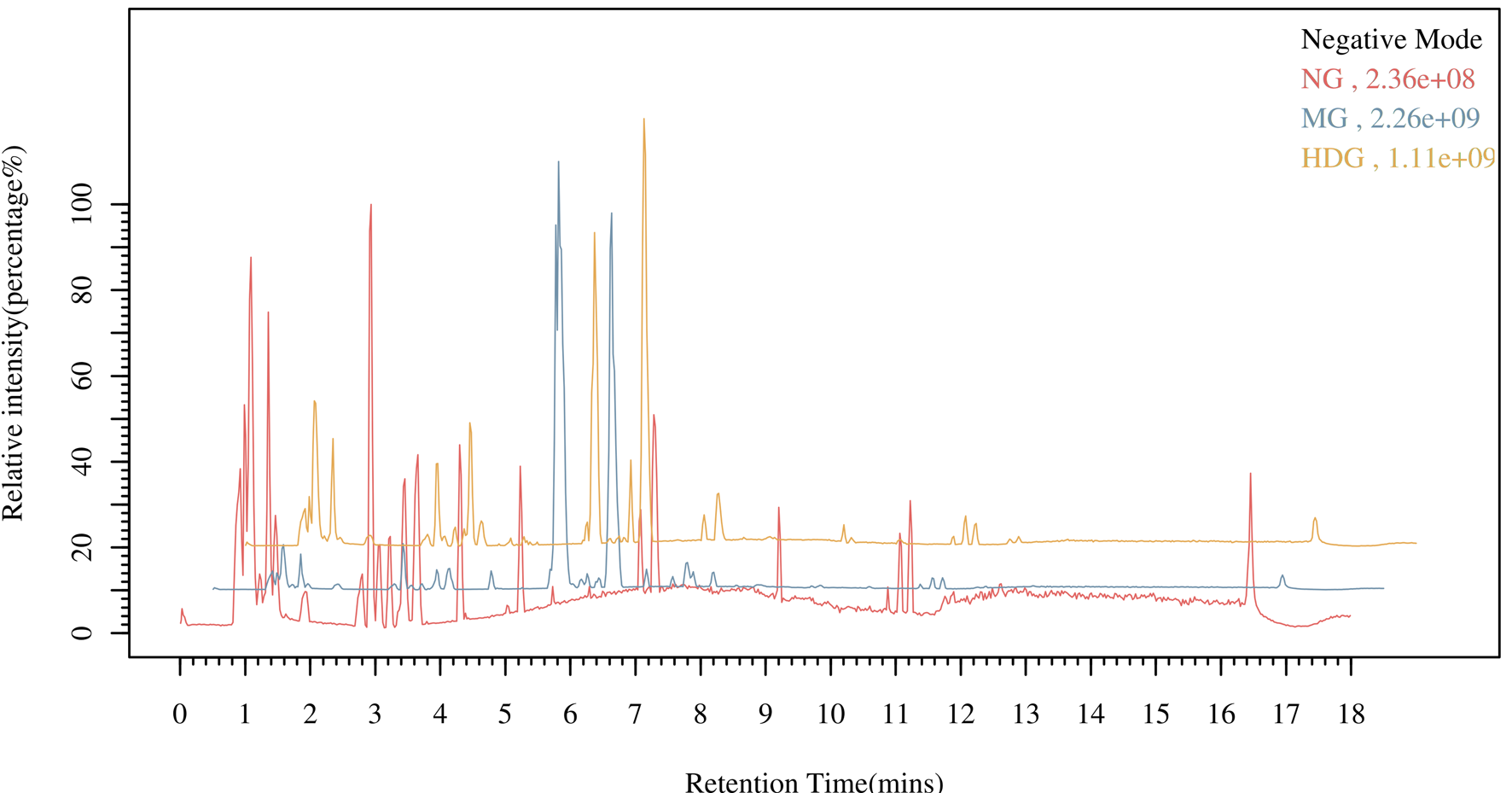


**Figure S5** Base peak chromatograms of typical serum sample in negative mode.


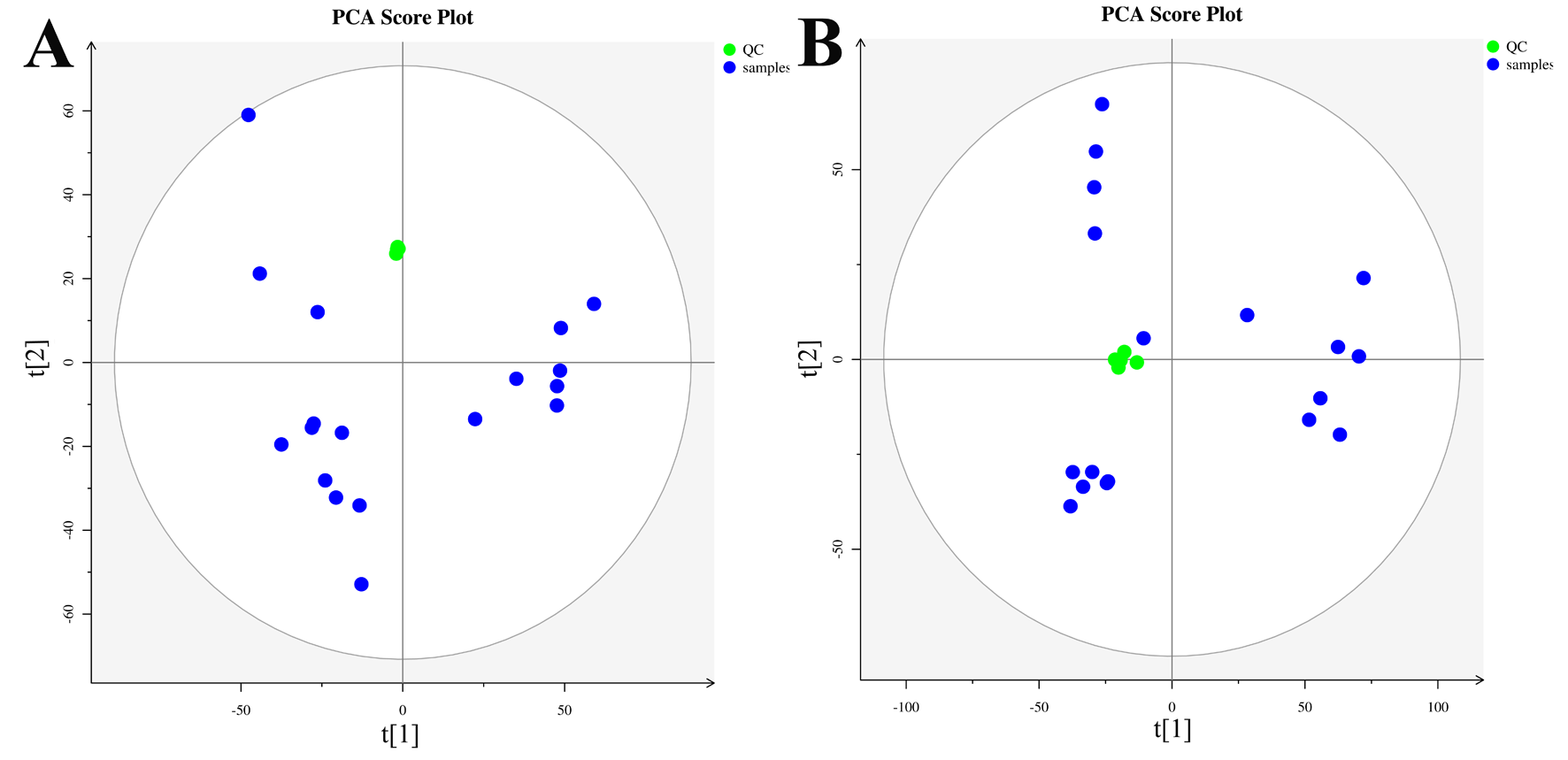


**Figure S6** Principal Component Analysis (PCA) Score plot of quality control (QC) Samples at positive ion mode(A) and negative ion mode(B).

1.2 Supplementary Tables

Table S1 Mass spectrometry qualitative results of HGJD by by UPLC-Q-Exactive MS/MS

| NO. | Compound | RT  (min) | Formula | Precursor ion | Predictived | Measured | Error  (ppm) | MS^2^ | Source | Reference |
| --- | --- | --- | --- | --- | --- | --- | --- | --- | --- | --- |
| 1 | Synephrine* | 1.44 | C_9_H_13_NO_2_ | [M+H]^+^ | 168.1019 | 168.1019 | 0.00 | 150.0914, 135.0679, 119.0494, 107.0495, 91.0548 | CP, QP | (Zheng et al., 2019) |
| 2  [S1] | Acetophenone | 1.88 | C_8_H_8_O | [M+H]^+^ | 121.0647 | 121.0652 | 4.13 | 103.0546, 91.0547, 53.0393 | CP | (Zheng et al., 2020) |
| 3  [S2] | Quinic acid | 1.94 | C_7_H_12_O_6_ | [M-H]^-^ | 191.0561 | 191.0559 | -1.05 | 173.0451, 85.0286 | ZZ | (Zhou et al., 2020) |
| 4 | Shikimic acid | 2.01 | C_7_H_10_O_5_ | [M-H]^-^ | 173.0455 | 173.0452 | -1.73 | 137.0238 | ZZ | (Zhou et al., 2020) |
| 5  [S3] | Nicotinamide | 2.46 | C_6_H_6_N_2_O | [M+H]^+^ | 123.0552 | 123.0556 | 3.25 | 88.0237, 80.0501, 56.9655, 53.0393 | CP | (Zheng et al., 2020) |
| 6 | Nicotinic acid | 2.57 | C_6_H_5_NO_2_ | [M+H]^+^ | 124.0393 | 124.0396 | 2.42 | 96.0449, 80.0504, 78.0345, 53.0393 | CP, QP | (Zheng et al., 2020) |
| 7  [S4] | Citric acid | 3.23 | C_6_H_8_O_7_ | [M-H]^-^ | 191.0197 | 191.0196 | -0.52 | 111.0080, 87.0079 | BS | (Xu et al., 2018) |
| 8 | Gallic acid* | 4.29 | C_7_H_6_O_5_ | [M-H]^-^ | 169.0142 | 169.0139 | -1.77 | 125.0237, 97.0287 | BS, MDP | (Li et al., 2018; Qi et al., 2014) |
| 9  [S5] | Geniposidic acid | 4.67 | C_16_H_22_O_10_ | [M-H]^-^ | 373.114 | 373.1147 | 1.88 | 211.0613, 167.0708, 123.0444, 149.0602 | ZZ | (Wang et al., 2016) |
| 10  [S6] | Shanzhiside | 4.71 | C_16_H_24_O_11_ | [M-H]^-^ | 391.1245 | 391.1255 | 2.56 | 229.0716, 211.0614, 193.0507, 185.0818 | ZZ | (Zhou et al., 2020) |
| 11  [S7] | Gardenoside | 5.64 | C_17_H_24_O_11_ | [M-H]^-^ | 403.1245 | 403.1257 | 2.98 | 241.0719, 223.0609 | ZZ | (Fu et al., 2014) |
| 12  [S8] | Jasminoside B | 6.47 | C_16_H_26_O_8_ | [M+H]^+^ | 347.1700 | 347.1703 | 0.86 | 185.1172, 167.1067 | ZZ | (Zhou et al., 2020) |
| 13 | Neochlorogenic acid | 6.55 | C_16_H_18_O_9_ | [M+H]+/  [M-H]- | 355.1023/ 353.0878 | 355.1025/ 353.0887 | 0.56/  2.55 | 163.0390, 135.0441/191.0559 | ZZ | (Zhou et al., 2020) |
| 14  [S9] | Methyl gallate | 6.63 | C_8_H_8_O_5_ | [M-H]^-^ | 183.0298 | 183.0298 | 0.00 | 168.0060, 124.0159 | MDP, BS | (Li et al., 2018) |
| 15 | Procyanidin B3 | 6.63 | C_30_H_26_O_12_ | [M-H]^-^ | 577.1351 | 577.1366 | 2.60 | 425.0886, 407.0773, 289.0723, 245.0821, 203.0712, 161.0240, 137.0238, 125.0238, 109.0287 | MDP | (Zhan et al., 2018) |
| 16  [S10] | Oxypaeoniflorin | 7.02 | C_23_H_28_O_12_ | [M-H]^-^ | 495.1508 | 495.1516 | 1.62 | 465.1408, 333.1000, 165.0555, 137.0238, 93.0338 | BS, MDP | (Zhan et al., 2018) |
| 17 | Isomers of procyanidin B3 | 7.2 | C_30_H_26_O_12_ | [M-H]^-^ | 577.1351 | 577.1375 | 4.16 | 425.0886, 289.0723, 245.0821, 203.0712, 137.0238, 109.0287 | MDP | (Zhan et al., 2018) |
| 18 | Mudanpioside E | 7.40 | C_24_H_29_O_13_ | [M-H]^-^ | 525.1613 | 525.1624 | 2.09 | 167.0345, 123.0444, 495.1535 | MDP | (Zhan et al., 2018) |
| 19 | Catechin | 7.6 | C_15_H_14_O_6_ | [M-H]^-^ | 289.0717 | 289.0724 | 2.42 | 245.0822, 137.0241, 123.0445, 109.0288 | BS | (Xiang et al., 2016; Xu et al., 2018) |
| 20 | p-Hydroxybenzoic acid | 7.61 | C_7_H_6_O_3_ | [M+H]+/  [M-H]^-^ | 139.0389/ 137.0244 | 139.039/ 137.0238 | 0.72/  -4.38 | 121.0399, 65.0393/93.0337 | MDP | (Du et al., 2019) |
| 21  [S11] | Chlorogenic acid* | 7.66 | C_16_H_18_O_9_ | [M+H]^+^ | 355.1023/ 353.0878 | 355.1023/ 353.0885 | 0/1.98 | 163.0389, 135.0441/191.0559 | ZZ | (Wang et al., 2016; Zhou et al., 2020) |
| 22 | Cryptochlorogenic acid | 7.69 | C_16_H_18_O_9_ | [M+H]^+^ | 355.1023 | 355.1024 | 0.28 | 163.0389, 137.0597 | ZZ | (Zhou et al., 2020) |
| 23 | Sipeimine-3-β-D-  glucoside | 7.73 | C_33_H_53_NO_8_ | [M+H]^+^ | 592.3843 | 592.3848 | 0.84 | 574.3735, 412.3208, 394.3105, 98.0969 | ZBM | (Zhou et al., 2013) |
| 24 | Kaempferol-3-rutinoside | 7.88 | C_27_H_30_O_15_ | [M+H]+/[M-H]^-^ | 595.1657/ 593.1512 | 595.1656/ 593.1522 | -0.17/1.69 | 121.0285, 287.0580/  255.0301, 284.0330, 285.0409 | ZZ | (Zhou et al., 2018) |
| 25 | Vicenin-2 | 7.88 | C_27_H_30_O_15_ | [M+H]^+^ | 595.1657 | 595.1656 | -0.17 | 389.1058, 164.5757 | CP, QP | (Luo et al., 2019) |
| 26  [S12] | Geniposide* | 8.01 | C_17_H_24_O_10_ | [M+H]^+^ | 389.1442 | 389.1437 | -1.28 | 191.0714 | ZZ | (Zhou et al., 2020) |
| 27  [S13] | Peimisine | 8.27 | C_27_H_41_NO_3_ | [M+H]^+^ | 428.3159 | 428.316 | 0.23 | 84.0814, 81.0705, 79.0547, 67.0549 | ZBM | (Zhou et al., 2013) |
| 28  [S14] | Peimine* | 8.35 | C_27_H_45_NO_3_ | [M+H]^+^ | 432.3472 | 432.3469 | -0.69 | 414.3365, 398.3051, 98.0969 | ZBM | (Wang et al., 2014) |
| 29 | Caffeic acid | 8.70 | C_9_H_8_O_4_ | [M-H]^-^ | 179.0349 | 179.0347 | -1.12 | 135.0445 | CP, ZZ | (Fu et al., 2014; Zheng et al., 2019) |
| 30  [S15] | Peiminine* | 8.71 | C_27_H_43_NO_3_ | [M+H]^+^ | 430.3315 | 430.3314 | -0.23 | 412.3209, 98.0968 | ZBM | (Zhou et al., 2013) |
| 31  [S16] | Albiflorin* | 8.78 | C_23_H_28_O_11_ | [M+H]+/[M+HCOO]^-^ | 481.1704/ 525.1613 | 481.1703/ 525.1619 | -0.21/1.14 | 319.1167, 197.0809, 133.0650/357.1197, 121.0288 | BS | (Qi et al., 2014; Xiang et al., 2016) |
| 32  [S17] | Paeoniflorin* | 9.23 | C_23_H_28_O_11_ | [M-H]^-^ | 525.1613 | 525.1625 | 2.29 | 449.1466, 327.1079, 165.0550, 121.0300 | BS, MDP | (Qi et al., 2014) |
| 33 | Eriocitrin | 9.78 | C_27_H_32_O_15_ | [M-H]^-^ | 595.1668 | 595.1683 | 2.52 | 287.0565, 151.0032, 135.0445 | CP, QP | (Xu et al., 2018) |
| 34 | Rutin* | 9.88 | C_27_H_30_O_16_ | [M-H]^-^ | 609.1461 | 609.1474 | 2.13 | 301.0357, 271.0253, 255.0302 | ZZ, CP | (Wang et al., 2016; Zheng et al., 2019) |
| 35  [S18] | Ebeiedinone | 10.32 | C_27_H_43_NO_2_ | [M+H]^+^ | 414.3366 | 414.3372 | 1.45 | 98.0969, 67.0550 | ZBM | (Zhou et al., 2013) |
| 36  [S19] | Isoquercitrin | 10.48 | C_21_H_20_O_12_ | [M+H]+/[M-H]^-^ | 465.1027/ 463.0881 | 465.1027/ 463.0895 | 0/3.02 | 303.0497/301.0722, 151.0032 | CP, ZZ | (Fu et al., 2014; Xu et al., 2018) |
| 37  [S20] | Puqiedinone | 10.63 | C_27_H_43_NO_2_ | [M+H]^+^ | 414.3366 | 414.3366 | 0 | 91.6237, 67.4471 | ZBM | (Zhou et al., 2013) |
| 38 | Naringenin-7-O-β-D-glucopyranoside | 10.88 | C_21_H_22_O_10_ | [M+H]^+^ | 435.1285 | 435.1283 | -0.46 | 273.0755, 153.0182, 147.0440 | BS | (Xu et al., 2018) |
| 39  [S21] | Narirutin* | 10.9 | C_27_H_32_O_14_ | [M+H]+/[M-H]^-^ | 581.1864/ 579.1719 | 581.1862/ 579.1724 | -0.34/0.86 | 273.0757, 153.0182/ 271.0616, 151.0032 | CP, QP | (Zheng et al., 2019) |
| 40 | Rhoifolin | 10.91 | C_27_H_30_O_14_ | [M+H]+/[M-H]^-^ | 579.1708/ 577.1562 | 579.171/ 577.1573 | 0.35/  1.91 | 433.1128, 271.0599/269.0459 | CP, QP | (Zheng et al., 2019) |
| 41 | Imperialine | 10.98 | C_27_H_43_NO_3_ | [M+H]^+^ | 430.3315 | 430.3313 | -0.46 | 69. 0704, 67.0549, 412.3204, 79.0549 | ZBM | (Zhou et al., 2013) |
| 42 | Sipeimine | 10.98 | C_27_H_43_NO_3_ | [M+H]^+^ | 430.3315 | 430.3315 | 0.00 | 412.3204, 67.0549 | ZBM | (Zhou et al., 2013) |
| 43 | Crocin* | 11.19 | C_44_H_64_O_24_ | [M-H]^-^ | 975.3714 | 975.3731 | 1.74 | 929.3364 | ZZ | (Fu et al., 2014) |
| 44 | Ferulic acid | 11.22 | C_10_H_10_O_4_ | [M-H]^-^ | 193.0506 | 193.0506 | 0.00 | 178.0268, 134.0367 | CP, QP | (Zheng et al., 2019) |
| 45 | Mudanpioside H | 11.27 | C_30_H_32_O_14_ | [M-H]^-^ | 615.1719 | 615.1746 | 4.39 | 431.1352, 281.0671, 169.0135 | MDP | (Zhan et al., 2018) |
| 46 | Naringin* | 11.28 | C_27_H_32_O_14_ | [M+H]+/[M-H]^-^ | 581.1864/ 579.1719 | 581.1848/ 579.1729 | -2.75/1.73 | 273.0757, 153.0182/  271.0616, 151.0032 | CP, QP | (Zheng et al., 2019) |
| 47  [S22] | Hesperetin | 11.43 | C_16_H_14_O_6_ | [M+H]^+^ | 303.0863 | 303.0859 | -1.32 | 285.0756, 219.0640, 201.0553, 177.0546, 171.0288, 153.0182, 135.0443, 117.0337, 89.0390, 67.0184 | CP, QP | (Zheng et al., 2020) |
| 48  [S23] | Galloylpaeoniflorin | 11.43 | C_30_H_32_O_15_ | [M+H]^+^ | 633.1814 | 633.1813 | -0.16 | 153.0184 | BS, MDP | (Xiang et al., 2016; Zhan et al., 2018) |
| 49  [S24] | Hesperidin* | 11.45 | C_28_H_34_O_15_ | [M+H]+/[M-H]^-^ | 611.1970/ 609.1824 | 611.1965/ 609.1832 | -0.82/1.31 | 303.0498, 153.0181/301.0723, 286.0487 | CP, QP | (Xu et al., 2018; Zheng et al., 2019) |
| 50 | Neohesperidin* | 11.8 | C_28_H_34_O_15_ | [M+H]+/[M-H]^-^ | 611.1970/ 609.1824 | 611.2007/ 609.184 | 6.05/  2.63 | 303.0862,153.0183/301.0722, 286.0486 | CP, QP | (Xu et al., 2018; Zheng et al., 2019) |
| 51 | Isochlorogenic acid B | 12.60 | C_25_H_24_O_12_ | [M+H]+/[M-H]^-^ | 517.1340/ 515.1194 | 517.1343/ 515.1204 | 0.58/  1.94 | 499.1243, 337.0917, 319.0822/  353.0884, 191.0559, 173.0452, 179.0347, 135.0445 | ZZ | (Zhou et al., 2020) |
| 52 | Poncirin | 12.75 | C_28_H_34_O_14_ | [M+H]+/[M-H]^-^ | 595.2021/ 593.1875 | 595.2021/ 593.1893 | 0/33.03 | 287.0912, 153.0182/285.0772 | CP, QP | (Zheng et al., 2019) |
| 53 | Mudanpioside C | 13.09 | C_30_H_32_O_13_ | [M-H]^-^ | 599.177 | 599.1788 | 3.00 | 431.1350, 281.0669 | BS, MDP | (Xu et al., 2018) |
| 54 | Benzoyloxypaeoniflorin | 13.60 | C_30_H_32_O_13_ | [M-H]^-^ | 599.177 | 599.1783 | 2.17 | 137.0238 | BS, MDP | (Xu et al., 2018) |
| 55 | Isosakuranetin | 13.86 | C_16_H_14_O_5_ | [M+H]^+^ | 287.0914 | 287.0912 | -0.70 | 153.0185, 171.0287, 161.0596, 133.0641 | CP, QP | (Zheng et al., 2019) |
| 56  [S25] | Didymin* | 13.9 | C_28_H_34_O_14_ | [M+H]+/[M-H]^-^ | 595.2021/ 593.1875 | 595.2017/ 593.1885 | -0.67/1.69 | 449.1414, 287.0912/285.0772 | CP, QP | (Tong et al., 2018) |
| 57 | Quercetin* | 15.1 | C_15_H_10_O_7_ | [M-H]^-^ | 301.0353 | 301.0362 | 2.99 | 178.9984, 151.0032, 121.0288, 107.0130, 93.0337, 83.0131, 65.0023 | ZZ, MDP | (Fu et al., 2014; Li et al., 2018) |
| 58 | Benzoylalbiflorin | 15.47 | C_30_H_32_O_12_ | [M-H]^-^ | 629.1864 | 629.1888 | 3.81 | 553.1710, 121.0288 | BS | (Liu et al., 2015) |
| 59 | Mudanpioside B | 15.47 | C_31_H_34_O_14_ | [M-H]^-^ | 629.1876 | 629.1887 | 1.75 | 121.0288, 165. 0554, 431.1353, 553.1710 | MDP, BS | (Ren et al., 2019) |
| 60 | Benzoylpaeoniflorin* | 15.52 | C_30_H_32_O_12_ | [M-H]^-^ | 583.182 | 583.1824 | 0.69 | 165.0558, 121.0291 | BS | (Xiang et al., 2016) |
| 61  [S26] | Naringenin | 16.85 | C_15_H_12_O_5_ | [M-H]^-^ | 271.0611 | 271.0616 | 1.84 | 151.0031, 119.0495, 107.0130 | CP, QP | (Zheng et al., 2019) |
| 62 | 16-oxo-Alisol A | 17.87 | C_30_H_48_O_6_ | [M+H]^+^ | 505.3523 | 505.3522 | -0.20 | 487.3423, 469.3315, 451.3217, 415.2842, 397.2737, 353.2471 | ZX | (Li et al., 2017) |
| 63  [S27] | Paeonol* | 19.1 | C_9_H_10_O_3_ | [M+H]^+^ | 167.0702 | 167.0703 | 0.60 | 149.0598, 121.0650 | BS, MDP | (Xu et al., 2018) |
| 64 | Sinensetin* | 19.1 | C_20_H_20_O_7_ | [M+H]^+^ | 373.1281 | 373.1278 | -0.80 | 343.0811, 181.0138 | CP, QP | (Cai et al., 2019) |
| 65 | Alisol C | 19.35 | C_30_H_46_O_5_ | [M+H]^+^ | 487.3418 | 487.3419 | 0.21 | 469.3308, 415.2841, 397.2742, 379.2742 | ZX | (Zhao et al., 2015) |
| 66  [S28] | Nobiletin* | 20.71 | C_21_H_22_O_8_ | [M+H]^+^ | 403.1387 | 403.1385 | -0.50 | 403.1384, 388.1147, 373.0916 | CP, QP | (Cai et al., 2019) |
| 67 | 16-oxo-11-anhydro-Alisol A | 20.75 | C_30_H_46_O_5_ | [M+H]^+^ | 487.3418 | 487.3418 | 0.00 | 469.3315, 451.3205, 397.2623 | ZX | (Zhao et al., 2015) |
| 68 | 6-Demethoxytangeretin | 20.92 | C_19_H_18_O_6_ | [M+H]^+^ | 343.1176 | 343.1175 | -0.29 | 327.0865, 313.0705, 299.0911, 282.0888, 267.0651, 239.0703, 199.0233, 153.0178 | CP, QP | (Zheng et al., 2020) |
| 69  [S29] | Hepta-3* | 21.72 | C_22_H_24_O_9_ | [M+H]^+^ | 433.1493 | 433.1489 | -0.92 | 418.1259, 403.1022, 385.0914 | CP, QP | (Cai et al., 2019) |
| 70  [S30] | Tangeretin | 22.45 | C_20_H_20_O_7_ | [M+H]^+^ | 373.1281 | 373.1282 | 0.27 | 358.1053, 343.0812, 211.0237 | CP, QP | (Xu et al., 2018) |
| 71 | 5-O-Demethylnobiletin | 24.01 | C_20_H_20_O_8_ | [M+H]^+^ | 389.123 | 389.1229 | -0.26 | 374.0991, 359.0758, 341.0655, 331.0794, 316.0576 | CP, QP | (Zheng et al., 2020) |
| 72 | Alisol C 23-acetate | 24.46 | C_32_H_48_O_6_ | [M+H]^+^ | 529.3523 | 529.3524 | 0.19 | 529.3521, 511.3400, 451.3205, 415.2840, 353.2486 | ZX | (Yang et al., 2020) |
| 73 | Alismaketone C 23-acetate | 24.46 | C_32_H_48_O_6_ | [M+H]^+^ | 529.3523 | 529.3524 | 0.19 | 469.3307, 397.2751 | ZX | (Yang et al., 2020) |
| 74  [S31] | Alisol B | 25.97 | C_30_H_48_O_4_ | [M+H]^+^ | 473.3625 | 473.3627 | 0.42 | 455.3521, 437.3420, 383.2944, 365.2838, 339.2680 | ZX | (Li et al., 2017) |
| 75 | Alisol B 23-acetate* | 33.47 | C_32_H_50_O_5_ | [M+H]^+^ | 515.3731 | 515.3727 | -0.78 | 497.3655, 479.3513, 455.3526, 437.3409, 419.3308, 339.2684 | ZX | (Li et al., 2017) |

CP: Citri Reticulatae Pericarpium (*Citrus reticulata* Blanco), QP: Citri Reticulatae Pericarpium Viride (*Citrus reticulata* Blanco), BS: Paeoniae Radix Alba (*Paeonia lactiflora* Pall.), MDP: Moutan Cortex (*Paeonia suffruticosa* Andr.), ZZ: Gardeniae Fructus (*Gardenia jasminoides* Ellis), ZX: Alismatis Rhizaoma (*Alisma plantago-aquatica* Linn.), ZBM: Fritillariae Thunbergii Bulbus (*Fritillaria thunbergii* Miq.).

The substances with * in the table have been compared with the reference substance.

[S1] to [S31] were the compounds that have also been detected in serum samples after oral administration of HGJD.

**Table S2** Information of anti-CHI targets of HGJD

| **Component** | **Target** | **Component** | **Target** | **Component** | **Target** |
| --- | --- | --- | --- | --- | --- |
| Acetophenone | LYZ | Alisol B | REN | Chlorogenic acid | SELE |
| Albiflorin | SOD2 | Alisol B | MMP2 | Chlorogenic acid | MAPK8 |
| Albiflorin | CYP2C9 | Alisol B | ADK | Chlorogenic acid | GSTM1 |
| Albiflorin | REN | Alisol B | PPARA | Chlorogenic acid | PLAU |
| Albiflorin | NR1H3 | Alisol B | NR1H2 | Chlorogenic acid | MMP2 |
| Albiflorin | MAPK14 | Alisol B | IGF1 | Citric acid | SELE |
| Albiflorin | SRC | Alisol B | S100A9 | Citric acid | HMGCR |
| Albiflorin | HMOX1 | Alisol B | CES2 | Citric acid | RNASE3 |
| Albiflorin | XIAP | Alisol B | HMGCR | Citric acid | CAT |
| Albiflorin | PIK3CG | Alisol B | NR1H3 | Citric acid | MME |
| Albiflorin | SULT2A1 | Alisol B | CYP17A1 | Citric acid | OTC |
| Albiflorin | ELANE | Alisol B | CYP19A1 | Citric acid | CTSG |
| Albiflorin | NR1I2 | Alisol B | PTGS2 | Citric acid | G6PD |
| Albiflorin | ADK | Alisol B | NR1I2 | Citric acid | HRAS |
| Albiflorin | LGALS3 | Alisol B | PRKCA | Citric acid | GSR |
| Albiflorin | ABCB1 | Alisol B | PYGL | Citric acid | CASP3 |
| Albiflorin | PRKCA | Alisol B | HSD11B2 | Citric acid | GSTM1 |
| Albiflorin | SELP | Alisol B | CNR1 | Citric acid | PKLR |
| Albiflorin | MMP7 | Alisol B | AR | Citric acid | AGXT |
| Albiflorin | HSPA8 | Alisol B | MAPK8 | Citric acid | F10 |
| Albiflorin | GBA | Alisol B | KIT | Citric acid | PTGS2 |
| Albiflorin | VEGFA | Alisol B | KDR | Citric acid | SRC |
| Albiflorin | FDFT1 | Alisol B | MAPK14 | Citric acid | GRIN2A |
| Albiflorin | NRAS | Alisol B | MAP3K14 | Didymin | STAT1 |
| Albiflorin | AGTR1 | Alisol B | MAPK1 | Didymin | PKLR |
| Albiflorin | SERPINE1 | Alisol B | VHL | Didymin | IGF1 |
| Albiflorin | F3 | Alisol B | CHUK | Didymin | LYZ |
| Albiflorin | HRAS | Alisol B | PYGM | Didymin | KIT |
| Albiflorin | IGFBP3 | Alisol B | F10 | Didymin | HRAS |
| Albiflorin | MMP3 | Alisol B | MMP3 | Didymin | SERPINA1 |
| Albiflorin | MMP9 | Alisol B | CETP | Didymin | MMP9 |
| Albiflorin | MME | Alisol B | NR3C2 | Didymin | LGALS3 |
| Alisol B | IL2 | Chlorogenic acid | LYZ | Didymin | CCL5 |
| Alisol B | ELANE | Chlorogenic acid | INSR | Didymin | ACADM |
| Alisol B | VDR | Chlorogenic acid | RNASE3 | Didymin | ESR1 |
| Alisol B | CYP2C8 | Chlorogenic acid | PKLR | Didymin | PTGS2 |
| Alisol B | NR1H4 | Chlorogenic acid | AGXT | Didymin | RXRA |
| Alisol B | FABP6 | Chlorogenic acid | ALB | Didymin | PIK3CG |
| Alisol B | FECH | Chlorogenic acid | SULT2B1 | Didymin | NCOA1 |
| Alisol B | GSTP1 | Chlorogenic acid | MMP9 |  |  |

**Table S2** *(continued)* Information of anti-CHI targets of HGJD

| **Component** | **Target** | **Component** | **Target** | **Component** | **Target** |
| --- | --- | --- | --- | --- | --- |
| Ebeiedinone | IGF1 | Gardenoside | HRAS | Hepta-3 | CA2 |
| Ebeiedinone | KIT | Gardenoside | MMP9 | Hepta-3 | APP |
| Ebeiedinone | SULT2B1 | Gardenoside | NOS2 | Hepta-3 | CYP1B1 |
| Ebeiedinone | ALB | Gardenoside | RNASE3 | Hepta-3 | KIT |
| Ebeiedinone | VDR | Gardenoside | ELANE | Hepta-3 | SRC |
| Ebeiedinone | RXRA | Gardenoside | MME | Hepta-3 | ABCB1 |
| Ebeiedinone | PPARA | Gardenoside | RNASE2 | Hepta-3 | KDR |
| Ebeiedinone | NR1H3 | Gardenoside | SELE | Hepta-3 | PIK3CG |
| Ebeiedinone | IL2 | Gardenoside | GSTM1 | Hepta-3 | MET |
| Ebeiedinone | GSTP1 | Gardenoside | SULT2B1 | Hepta-3 | MCL1 |
| Ebeiedinone | F2 | Geniposide | HRAS | Hepta-3 | PTGS2 |
| Ebeiedinone | ESR1 | Geniposide | STAT1 | Hepta-3 | AURKB |
| Ebeiedinone | ADK | Geniposide | MMP9 | Hepta-3 | AXL |
| Ebeiedinone | ADH5 | Geniposide | PLAU | Hepta-3 | ALK |
| Ebeiedinone | BCHE | Geniposide | CES1 | Hepta-3 | GPR35 |
| Ebeiedinone | AR | Geniposide | AKT1 | Hepta-3 | AKT1 |
| Ebeiedinone | NR3C1 | Geniposide | MAN1B1 | Hepta-3 | XDH |
| Galloylpaeoniflorin | IGF1 | Geniposide | CA2 | Hepta-3 | AKR1C2 |
| Galloylpaeoniflorin | REN | Geniposide | BCL2 | Hepta-3 | AKR1C4 |
| Galloylpaeoniflorin | CYP2C9 | Geniposide | HMOX1 | Hepta-3 | MMP2 |
| Galloylpaeoniflorin | S100A9 | Geniposide | GCG | Hepta-3 | ABCC1 |
| Galloylpaeoniflorin | PIK3R1 | Geniposide | GSTM1 | Hepta-3 | MMP9 |
| Galloylpaeoniflorin | IL2 | Geniposidic acid | MME | Hepta-3 | NOS2 |
| Galloylpaeoniflorin | SERPINA1 | Geniposidic acid | SRC | Hepta-3 | INSR |
| Galloylpaeoniflorin | NR1H3 | Geniposidic acid | NOS2 | Hepta-3 | MMP3 |
| Galloylpaeoniflorin | XIAP | Geniposidic acid | MMP9 | Hepta-3 | CYP19A1 |
| Galloylpaeoniflorin | ELANE | Geniposidic acid | OTC | Hepta-3 | ARG1 |
| Galloylpaeoniflorin | ALB | Geniposidic acid | HRAS | Hepta-3 | PYGL |
| Galloylpaeoniflorin | VDR | Geniposidic acid | CA2 | Hepta-3 | FLT4 |
| Galloylpaeoniflorin | TGFB2 | Geniposidic acid | F2 | Hepta-3 | MAPK9 |
| Galloylpaeoniflorin | KIT | Hepta-3 | ELANE | Hepta-3 | ESR1 |
| Galloylpaeoniflorin | RARA | Hepta-3 | F2 | Hepta-3 | AR |
| Galloylpaeoniflorin | MMP2 | Hepta-3 | GBA | Hepta-3 | F10 |
| Galloylpaeoniflorin | RBP4 | Hepta-3 | CASP3 | Hepta-3 | KCNMA1 |
| Galloylpaeoniflorin | PPARA | Hepta-3 | ESR2 |  |  |
| Galloylpaeoniflorin | SOD2 | Hepta-3 | SHBG |  |  |
| Galloylpaeoniflorin | FECH | Hepta-3 | ADH5 |  |  |
| Galloylpaeoniflorin | NR1I3 | Hepta-3 | BACE1 |  |  |
| Galloylpaeoniflorin | ACADM | Hepta-3 | MAPK8 |  |  |
| Gardenoside | STAT1 | Hepta-3 | ABCG2 |  |  |

**Table S2 *(continued)* Information of anti-CHI targets of HGJD**

| **Component** | **Target** | **Component** | **Target** | **Component** | **Target** |
| --- | --- | --- | --- | --- | --- |
| Hesperetin | GSR | Isoquercitrin | XIAP | Naringenin | CYP19A1 |
| Hesperetin | SRC | Isoquercitrin | VDR | Naringenin | ABCC1 |
| Hesperetin | Esr2 | Isoquercitrin | STAT1 | Naringenin | SHBG |
| Hesperetin | SHBG | Isoquercitrin | PYGL | Naringenin | CYP1B1 |
| Hesperetin | SERPINA1 | Isoquercitrin | PIK3R1 | Naringenin | MAOB |
| Hesperetin | NR1I2 | Isoquercitrin | MET | Naringenin | ABCG2 |
| Hesperetin | ADH5 | Isoquercitrin | LYZ | Naringenin | PTGS2 |
| Hesperetin | VDR | Isoquercitrin | LGALS3 | Naringenin | PIK3CG |
| Hesperetin | MMP3 | Isoquercitrin | INSR | Naringenin | RELA |
| Hesperetin | GSTM1 | Isoquercitrin | GSR | Naringenin | AKT1 |
| Hesperetin | CYP1B1 | Isoquercitrin | CES1 | Naringenin | BCL2 |
| Hesperetin | CYP19A1 | Isoquercitrin | CA2 | Naringenin | MAPK3 |
| Hesperetin | ABCG2 | Isoquercitrin | PTGS2 | Naringenin | MAPK1 |
| Hesperetin | ESR1 | Jasminoside B | PKLR | Naringenin | CASP3 |
| Hesperetin | ABCC1 | Jasminoside B | LYZ | Naringenin | LDLR |
| Hesperetin | MAOB | Jasminoside B | FABP6 | Naringenin | SOD1 |
| Hesperetin | PTGS2 | Jasminoside B | ACADM | Naringenin | PPARG |
| Hesperetin | PIK3CG | Jasminoside B | SELE | Naringenin | MTTP |
| Hesperetin | NCOA1 | Jasminoside B | LGALS3 | Naringenin | APOB |
| Hesperidin | SRC | Jasminoside B | MAPK1 | Naringenin | HMGCR |
| Hesperidin | LYZ | Jasminoside B | FECH | Naringenin | UGT1A1 |
| Hesperidin | CCL5 | Jasminoside B | HRAS | Naringenin | PPARA |
| Hesperidin | KIT | Jasminoside B | SERPINA1 | Naringenin | ADIPOQ |
| Hesperidin | HRAS | Jasminoside B | NOS2 | Naringenin | CES1 |
| Hesperidin | RNASE3 | Methyl gallate | CA2 | Narirutin | CCL5 |
| Hesperidin | MMP9 | Methyl gallate | PTGS2 | Narirutin | HRAS |
| Hesperidin | SULT2B1 | Methyl gallate | MAOB | Narirutin | STAT1 |
| Hesperidin | ELANE | Naringenin | GSR | Narirutin | LYZ |
| Hesperidin | HMGCR | Naringenin | VDR | Narirutin | KIT |
| Hesperidin | AGXT | Naringenin | ADH5 | Narirutin | SULT2B1 |
| Hesperidin | SHBG | Naringenin | NOS3 | Narirutin | PKLR |
| Hesperidin | RNASE2 | Naringenin | CA2 | Narirutin | CYP2C9 |
| Hesperidin | CYP19A1 | Naringenin | INSR | Narirutin | NR1I2 |
| Hesperidin | CASP3 | Naringenin | BCHE | Narirutin | MMP9 |
| Hesperidin | PTGS2 | Naringenin | KDR | Narirutin | GSR |
| Hesperidin | ICAM1 | Naringenin | YARS1 | Narirutin | SHBG |
| Hesperidin | VCAM1 | Naringenin | GBA | Narirutin | RNASE2 |
| Isoquercitrin | SULT2B1 | Naringenin | ESR1 | Narirutin | CYP19A1 |
| Isoquercitrin | KIT | Naringenin | F2 |  |  |
| Isoquercitrin | ELANE | Naringenin | ESR2 |  |  |

**Table S2** *(continued)* Information of anti-CHI targets of HGJD

| **Component** | **Target** | **Component** | **Target** | **Component** | **Target** |
| --- | --- | --- | --- | --- | --- |
| Nicotinamide | CA2 | Oxypaeoniflorin | VDR | Peimine | ALB |
| Nicotinamide | XIAP | Paeoniflorin | ELANE | Peimine | STS |
| Nicotinamide | BACE1 | Paeoniflorin | XIAP | Peimine | MME |
| Nicotinamide | PTGS2 | Paeoniflorin | ALB | Peimine | FECH |
| Nicotinamide | LYZ | Paeoniflorin | VDR | Peimine | DPP4 |
| Nobiletin | SHBG | Paeoniflorin | TTPA | Peimine | MMP3 |
| Nobiletin | GBA | Paeoniflorin | LGALS3 | Peimine | BMP2 |
| Nobiletin | RXRA | Paeoniflorin | ABCB1 | Peimine | PYGL |
| Nobiletin | RNASE3 | Paeoniflorin | VEGFA | Peimine | TTR |
| Nobiletin | AKT1 | Paeoniflorin | F10 | Peimine | CA2 |
| Nobiletin | HRAS | Paeoniflorin | SERPINE1 | Peimine | CYP2C9 |
| Nobiletin | ABCG2 | Paeoniflorin | BACE1 | Peimine | NR1H2 |
| Nobiletin | CYP1B1 | Paeoniflorin | SELP | Peimine | SULT2A1 |
| Nobiletin | KIT | Paeoniflorin | TNF | Peimine | GBA |
| Nobiletin | NOS2 | Paeoniflorin | IL6 | Peiminine | F2 |
| Nobiletin | F2 | Paeoniflorin | CD14 | Peiminine | TGFB2 |
| Nobiletin | ESR1 | Paeonol | HSPA8 | Peiminine | IGF1 |
| Nobiletin | AR | Paeonol | IL2 | Peiminine | RARA |
| Nobiletin | PPARG | Paeonol | F10 | Peiminine | SULT2B1 |
| Nobiletin | F10 | Paeonol | BCHE | Peiminine | BMP2 |
| Nobiletin | PTGS2 | Paeonol | MAPK14 | Peiminine | PPARG |
| Nobiletin | ESR2 | Paeonol | GBA | Peiminine | RXRA |
| Nobiletin | DPP4 | Paeonol | PYGL | Peiminine | STS |
| Nobiletin | KCNMA1 | Paeonol | STAT1 | Peiminine | BCHE |
| Nobiletin | BCL2 | Paeonol | RNASE3 | Peiminine | AR |
| Nobiletin | CASP9 | Paeonol | HMGCR | Peimisine | RARA |
| Nobiletin | MMP9 | Paeonol | CA2 | Peimisine | SHBG |
| Nobiletin | TP53 | Paeonol | MAOB | Peimisine | HMGCR |
| Nobiletin | MAPK8 | Paeonol | KCNMA1 | Peimisine | RBP4 |
| Nobiletin | TIMP1 | Paeonol | PTGS2 | Peimisine | PPARG |
| Nobiletin | CREB1 | Paeonol | RELA | Peimisine | VDR |
| Oxypaeoniflorin | NR1H4 | Paeonol | AKT1 | Peimisine | ADH5 |
| Oxypaeoniflorin | F10 | Paeonol | BCL2 | Peimisine | AKR1C2 |
| Oxypaeoniflorin | MAPK1 | Paeonol | MAPK1 | Peimisine | ALB |
| Oxypaeoniflorin | NR1H3 | Paeonol | TNF | Peimisine | CA2 |
| Oxypaeoniflorin | HMOX1 | Paeonol | NFKBIA | Peimisine | ADK |
| Oxypaeoniflorin | CYP2C9 | Paeonol | ICAM1 | Peimisine | MET |
| Oxypaeoniflorin | XIAP | Peimine | IGF1 | Peimisine | SHH |
| Oxypaeoniflorin | REN | Peimine | PPARA | Peimisine | EBP |
| Oxypaeoniflorin | ELANE | Peimine | TGFB2 | Peimisine | NR3C2 |
| Oxypaeoniflorin | SOD2 | Peimine | PIK3R1 | Peimisine | NR3C1 |

**Table S2** *(continued)* Information of anti-CHI targets of HGJD

| **Component** | **Target** | **Component** | **Target** | **Component** | **Target** |
| --- | --- | --- | --- | --- | --- |
| Puqiedinone | PPARA | Quinic acid | RAC1 | Tangeretin | MMP3 |
| Puqiedinone | GSTP1 | Quinic acid | FDFT1 | Tangeretin | ESR2 |
| Puqiedinone | RXRA | Quinic acid | PTGS2 | Tangeretin | NOS3 |
| Puqiedinone | VDR | Shanzhiside | REN | Tangeretin | RXRA |
| Puqiedinone | IGF1 | Shanzhiside | SELE | Tangeretin | GSR |
| Puqiedinone | IL2 | Shanzhiside | NOS2 | Tangeretin | ABCG2 |
| Puqiedinone | F2 | Shanzhiside | HRAS | Tangeretin | KIT |
| Puqiedinone | NR1H3 | Shanzhiside | ADK | Tangeretin | CYP1B1 |
| Puqiedinone | ADK | Shanzhiside | MMP3 | Tangeretin | NOS2 |
| Puqiedinone | ADH5 | Shanzhiside | GALK1 | Tangeretin | PTGS2 |
| Puqiedinone | KIT | Shanzhiside | RNASE2 | Tangeretin | F2 |
| Puqiedinone | ESR1 | Shanzhiside | RHOA | Tangeretin | AR |
| Puqiedinone | SULT2B1 | Tangeretin | ESR1 | Tangeretin | DPP4 |
| Puqiedinone | ALB | Tangeretin | HRAS | Tangeretin | KCNMA1 |
| Puqiedinone | BCHE | Tangeretin | MAPK14 | Tangeretin | CDKN1A |
| Quinic acid | ACE | Tangeretin | F10 | Tangeretin | HMOX1 |
| Quinic acid | F10 | Tangeretin | SHBG | Tangeretin | CYP1A1 |
| Quinic acid | PYGL | Tangeretin | NR1I2 |  |  |

**Table S3** KEGG pathways enrichment analysis of potential targets for HGJD treatment of CHI (Top 20)

| **ID** | **Term** | **Gene** | **Count** | **Gene ratio** | ***P*Value** |
| --- | --- | --- | --- | --- | --- |
| hsa05200 | Pathways in cancer | CDKN1A, GSTP1, XIAP, PIK3R1, PTGS2, RELA, PIK3CG, CASP9, MAPK9, SHH, NRAS, MAPK8, RXRA, CASP3, AKT1, MAPK1, VHL, RAC1, HRAS, MAPK3, TGFB2, NOS2, CHUK, STAT1, MMP2, PRKCA, IGF1, MMP9, RHOA, VEGFA, NFKBIA, AR, BMP2, IL6, KIT, BCL2, RARA, AGTR1, PPARG, MET, TP53 | 41 | 0.26 | 5.26×10^-18^ |
| hsa04151 | PI3K-Akt signaling pathway | CDKN1A, FLT4, PIK3R1, RELA, PIK3CG, CASP9, NRAS, RXRA, KDR, AKT1, MAPK1, RAC1, HRAS, MCL1, MAPK3, CHUK, NOS3, INSR, PRKCA, IGF1, IL2, VEGFA, IL6, CREB1, KIT, BCL2, MET, TP53 | 28 | 0.18 | 1.51×10^-09^ |
| hsa05161 | Hepatitis B | CDKN1A, SRC, PIK3R1, TNF, RELA, PIK3CG, CASP9, MAPK9, NRAS, MAPK8, CASP3, AKT1, MAPK1, HRAS, MAPK3, TGFB2, CHUK, STAT1, PRKCA, MMP9, NFKBIA, IL6, CREB1, BCL2, TP53 | 25 | 0.16 | 1.19E-15 |
| hsa05205 | Proteoglycans in cancer | CDKN1A, SRC, PIK3R1, TNF, PIK3CG, NRAS, PLAU, CASP3, KDR, AKT1, MAPK1, RAC1, HRAS, MAPK3, TGFB2, MMP2, PRKCA, IGF1, MAPK14, MMP9, ESR1, RHOA, VEGFA, MET, TP53 | 25 | 0.16 | 1.85×10^-12^ |
| hsa04668 | TNF signaling pathway | VCAM1, CHUK, MMP3, PIK3R1, PTGS2, MAPK14, SELE, TNF, MMP9, PIK3CG, RELA, ICAM1, NFKBIA, MAPK9, IL6, MAPK8, CREB1, CASP3, CCL5, AKT1, MAPK1, MAP3K14, MAPK3 | 23 | 0.15 | 1.56×10^-16^ |
| hsa04014 | Ras signaling pathway | CHUK, FLT4, INSR, PRKCA, PIK3R1, IGF1, RHOA, PIK3CG, RELA, VEGFA, MAPK9, GRIN2A, NRAS, MAPK8, KIT, KDR, AKT1, MAPK1, RAC1, HRAS, MET, MAPK3 | 22 | 0.14 | 6.13×10^-09^ |
| hsa05160 | Hepatitis C | CDKN1A, CHUK, STAT1, NR1H3, PIK3R1, MAPK14, TNF, PIK3CG, RELA, NFKBIA, MAPK9, NRAS, MAPK8, RXRA, AKT1, MAPK1, PPARA, HRAS, LDLR, TP53, MAPK3 | 21 | 0.13 | 2.24×10^-12^ |
| hsa04015 | Rap1 signaling pathway | SRC, FLT4, INSR, PRKCA, PIK3R1, IGF1, MAPK14, RHOA, PIK3CG, VEGFA, GRIN2A, NRAS, CNR1, KIT, KDR, AKT1, MAPK1, RAC1, HRAS, MET, MAPK3 | 21 | 0.13 | 9.58×10^-09^ |
| hsa05152 | Tuberculosis | TGFB2, NOS2, STAT1, SRC, VDR, MAPK14, TNF, RHOA, RELA, CASP9, MAPK9, IL6, MAPK8, CREB1, CASP3, BCL2, AKT1, MAPK1, CD14, MAPK3 | 20 | 0.13 | 3.16×10^-09^ |

**Table S3** (*continued*) KEGG pathways enrichment analysis of potential targets for HGJD treatment of CHI (Top 20)

| **ID** | **Term** | **Gene** | **Count** | **Gene ratio** | ***P*Value** |
| --- | --- | --- | --- | --- | --- |
| hsa04066 | HIF-1 signaling pathway | CDKN1A, NOS2, NOS3, INSR, SERPINE1, PRKCA, PIK3R1, IGF1, PIK3CG, RELA, VEGFA, IL6, BCL2, AKT1, HMOX1, MAPK1, VHL, TIMP1, MAPK3 | 19 | 0.12 | 6.16×10^-13^ |
| hsa05142 | Chagas disease | TGFB2, ACE, NOS2, CHUK, SERPINE1, PIK3R1, MAPK14, TNF, IL2, PIK3CG, RELA, NFKBIA, MAPK9, IL6, MAPK8, CCL5, AKT1, MAPK1, MAPK3 | 19 | 0.12 | 2.60×10^-12^ |
| hsa05145 | Toxoplasmosis | HSPA8, TGFB2, NOS2, CHUK, STAT1, XIAP, MAPK14, TNF, RELA, CASP9, NFKBIA, MAPK9, MAPK8, CASP3, BCL2, AKT1, MAPK1, LDLR, MAPK3 | 19 | 0.12 | 7.03×10^-12^ |
| hsa04071 | Sphingolipid signaling pathway | ABCC1, NOS3, PRKCA, PIK3R1, MAPK14, TNF, RHOA, PIK3CG, RELA, MAPK9, NRAS, MAPK8, BCL2, AKT1, MAPK1, RAC1, HRAS, TP53, MAPK3 | 19 | 0.12 | 3.21×10^-11^ |
| hsa04510 | Focal adhesion | SRC, FLT4, XIAP, PRKCA, PIK3R1, IGF1, RHOA, PIK3CG, VEGFA, MAPK9, MAPK8, KDR, BCL2, AKT1, MAPK1, RAC1, HRAS, MET, MAPK3 | 19 | 0.12 | 2.11×10^-07^ |
| hsa04010 | MAPK signaling pathway | HSPA8, TGFB2, CHUK, PRKCA, MAPK14, TNF, RELA, MAPK9, NRAS, MAPK8, CASP3, AKT1, MAPK1, CD14, RAC1, HRAS, TP53, MAP3K14, MAPK3 | 19 | 0.12 | 4.29×10^-06^ |
| hsa05166 | HTLV-I infection | CDKN1A, TGFB2, VCAM1, CHUK, XIAP, PIK3R1, TNF, IL2, PIK3CG, RELA, ICAM1, NFKBIA, IL6, NRAS, CREB1, AKT1, HRAS, TP53, MAP3K14 | 19 | 0.12 | 4.53×10^-06^ |
| hsa05206 | MicroRNAs in cancer | CDKN1A, TGFB2, ABCC1, ABCB1, PRKCA, PTGS2, MMP9, RHOA, VEGFA, NRAS, PLAU, CASP3, BCL2, CYP1B1, HMOX1, HRAS, MET, TP53, MCL1 | 19 | 0.12 | 2.33×10^-05^ |
| hsa04380 | Osteoclast differentiation | TGFB2, CHUK, STAT1, PIK3R1, MAPK14, TNF, PIK3CG, RELA, NFKBIA, MAPK9, MAPK8, CREB1, AKT1, MAPK1, PPARG, RAC1, MAP3K14, MAPK3 | 18 | 0.11 | 1.21×10^-09^ |
| hsa04068 | FoxO signaling pathway | CDKN1A, TGFB2, CHUK, INSR, PIK3R1, IGF1, MAPK14, SOD2, PIK3CG, MAPK9, IL6, NRAS, MAPK8, CAT, AKT1, MAPK1, HRAS, MAPK3 | 18 | 0.11 | 1.73×10^-09^ |
| hsa05164 | Influenza A | HSPA8, STAT1, PRKCA, PIK3R1, MAPK14, TNF, PIK3CG, RELA, ICAM1, CASP9, NFKBIA, MAPK9, IL6, MAPK8, CCL5, AKT1, MAPK1, MAPK3 | 18 | 0.11 | 9.31×10^-08^ |

**Table S4** GO enrichment analysis of the predicted targets for HGJD treatment of CHI (Top 10)

| **ID** | **Go term** | **Count** | **Classification** |
| --- | --- | --- | --- |
| GO:0045944 | positive regulation of transcription from RNA polymerase II promoter | 35 | Biological process |
| GO:0043066 | negative regulation of apoptotic process | 29 | Biological process |
| GO:0042493 | response to drug | 28 | Biological process |
| GO:0007165 | signal transduction | 27 | Biological process |
| GO:0045893 | positive regulation of transcription, DNA-templated | 24 | Biological process |
| GO:0055114 | oxidation-reduction process | 23 | Biological process |
| GO:0008284 | positive regulation of cell proliferation | 20 | Biological process |
| GO:0000122 | negative regulation of transcription from RNA polymerase II promoter | 20 | Biological process |
| GO:0001666 | response to hypoxia | 19 | Biological process |
| GO:0043065 | positive regulation of apoptotic process | 18 | Biological process |
| GO:0070062 | extracellular exosome | 64 | Cellular component |
| GO:0005886 | plasma membrane | 61 | Cellular component |
| GO:0005829 | cytosol | 60 | Cellular component |
| GO:0005634 | nucleus | 59 | Cellular component |
| GO:0005737 | cytoplasm | 55 | Cellular component |
| GO:0005615 | extracellular space | 54 | Cellular component |
| GO:0005576 | extracellular region | 44 | Cellular component |
| GO:0005654 | nucleoplasm | 38 | Cellular component |

**Table S4** (*continued*) GO enrichment analysis of the predicted targets for HGJD treatment of CHI (Top 10)

| **ID** | **Go term** | **Count** | **Classification** |
| --- | --- | --- | --- |
| GO:0016020 | membrane | 33 | Cellular component |
| GO:0005739 | mitochondrion | 28 | Cellular component |
| GO:0005515 | protein binding | 113 | Molecular function |
| GO:0008270 | zinc ion binding | 30 | Molecular function |
| GO:0042802 | identical protein binding | 29 | Molecular function |
| GO:0019899 | enzyme binding | 28 | Molecular function |
| GO:0005524 | ATP binding | 28 | Molecular function |
| GO:0042803 | protein homodimerization activity | 26 | Molecular function |
| GO:0003700 | transcription factor activity, sequence-specific DNA binding | 19 | Molecular function |
| GO:0043565 | sequence-specific DNA binding | 18 | Molecular function |
| GO:0046982 | protein heterodimerization activity | 17 | Molecular function |
| GO:0005102 | receptor binding | 16 | Molecular function |

**Table S5** Drug-Components-Targets network analysis of potential active components of HGJD

| **Name** | **Degree** | **Betweenness Centrality** | **Closeness Centrality** | **Neighborhood Connectivity** |
| --- | --- | --- | --- | --- |
| Hepta-3 | 45 | 0.1857 | 0.4216 | 5.3333 |
| Alisol B | 41 | 0.1804 | 0.4143 | 4.9756 |
| Naringenin | 38 | 0.1396 | 0.4090 | 5.2632 |
| Albiflorin | 32 | 0.1238 | 0.3987 | 5.0625 |
| Nobiletin | 27 | 0.0721 | 0.3906 | 6.8148 |
| Tangeretin | 24 | 0.0523 | 0.3859 | 7.1250 |
| Galloylpaeoniflorin | 23 | 0.0533 | 0.3843 | 6.0870 |
| Paeonol | 22 | 0.0521 | 0.3828 | 6.3182 |
| Hesperetin | 20 | 0.0373 | 0.3797 | 7.0500 |
| Peimine | 19 | 0.0487 | 0.3782 | 5.8947 |
| Hesperidin | 19 | 0.0385 | 0.3782 | 8.0000 |
| Citric acid | 19 | 0.0641 | 0.3782 | 6.4737 |
| Ebeiedinone | 18 | 0.0227 | 0.3767 | 7.8333 |
| Peimisine | 17 | 0.0468 | 0.3752 | 6.1176 |
| Isoquercitrin | 17 | 0.0244 | 0.3752 | 8.4118 |
| Didymin | 17 | 0.0268 | 0.3752 | 8.2353 |
| Puqiedinone | 16 | 0.0152 | 0.3738 | 8.3125 |
| Paeoniflorin | 16 | 0.0522 | 0.3738 | 5.8750 |
| Narirutin | 15 | 0.0173 | 0.3723 | 8.5333 |
| Chlorogenic acid | 14 | 0.0200 | 0.3709 | 7.2857 |
| Geniposide | 13 | 0.0340 | 0.3694 | 7.2308 |
| Peiminine | 12 | 0.0175 | 0.3680 | 7.0833 |
| Oxypaeoniflorin | 12 | 0.0124 | 0.3680 | 7.9167 |
| Jasminoside B | 12 | 0.0147 | 0.3680 | 7.5833 |
| Gardenoside | 12 | 0.0110 | 0.3680 | 9.0000 |
| Shanzhiside | 10 | 0.0268 | 0.3652 | 7.9000 |
| Geniposidic acid | 9 | 0.0082 | 0.3638 | 10.1111 |
| Quinic acid | 7 | 0.0244 | 0.3611 | 9.1429 |
| Nicotinamide | 6 | 0.0034 | 0.3597 | 12.0000 |
| Methyl gallate | 4 | 0.0011 | 0.3570 | 14.7500 |
| Acetophenone | 2 | 0.0003 | 0.3544 | 19.5000 |

**References**

Cai, H., Xu, Y., Xie, L., Duan, Y., Zhou, J., Liu, J., et al. (2019). Investigation on Spectrum-Effect Correlation between Constituents Absorbed into Blood and Bioactivities of Baizhu Shaoyao San before and after Processing on Ulcerative Colitis Rats by UHPLC/Q-TOF-MS/MS Coupled with Gray Correlation Analysis. *Molecules.* 24(5), 940. doi: 10.3390/molecules24050940

Du, X. G., Jiang, H. J., Zhang, H., and Yan, J. Z. (2019). Quality evaluation of Moutan Cortex Formula Granules. *China journal of chinese materia medica.* 44(10), 2065-2071. doi: 10.19540/j.cnki.cjcmm.20181224.002

Fu, Z. W., Ling, Y., Li, Z. X., Chen, M. C., Sun, Z. L., and Huang, C. G. (2014). HPLC-Q-TOF-MS/MS for analysis of major chemical constituents of Yinchen-Zhizi herb pair extract. *Biomed Chromatogr.* 28(4), 475-485. doi: 10.1002/bmc.3057

Li, C. R., Li, M. N., Yang, H., Li, P., and Gao, W. (2018). Rapid characterization of chemical markers for discrimination of Moutan Cortex and its processed products by direct injection-based mass spectrometry profiling and metabolomic method. *Phytomedicine.* 45, 76-83. doi: 10.1016/j.phymed.2018.04.003

Li, S., Jin, S., Song, C. W., Jia, S. L., Zhang, Y., Feng, Y. L., et al. (2017). The strategy for establishment of the multiple reaction monitoring based characteristic chemical profile of triterpenes in Alismatis rhizoma using two combined tandem mass spectrometers. *J. Chromatogr. A*. 1524, 121-134. doi: 10.1016/j.chroma.2017.09.057

Liu, J., Chen, L., Fan, C. R., Li, H., Huang, M. Q., Xiang, Q., et al. (2015). Qualitative and quantitative analysis of major constituents of Paeoniae Radix Alba and Paeoniae Radix Rubra by HPLC-DAD-Q-TOF-MS /MS. *China journal of chinese materia medica.* 40(9), 1762-1770. doi: 10.4268/cjcmm20150926

Luo, Y., Zeng, W., Huang, K.E., Li, D.X., Chen, W., Yu, X.Q., et al. (2019). Discrimination of Citrus reticulata Blanco and Citrus reticulata 'Chachi' as well as the Citrus reticulata 'Chachi' within different storage years using ultra high performance liquid chromatography quadrupole/time-of-flight mass spectrometry based metabolomics approach. *J. Pharm. Biomed. Anal.* 171, 218-231. doi: 10.1016/j.jpba.2019.03.056

Qi, Y., Li, S. Z., Pi, Z. F., Song, F. R., Lin, N., Liu, S., et al. (2014). Chemical profiling of Wu-tou decoction by UPLC-Q-TOF-MS. *Talanta.* 118, 21-29. doi: 10.1016/j.talanta.2013.09.054

Ren, J., Liu, X., Li, W. D., Cai, H., Zhu, X. C., and Cai, B. C. (2019). Qualitative Analysis of Major Constituents of Raw and Processed Paeonia Lactiflora by UHPLC-Q-TOF-MS /MS. *World chinese medicine.* 14(2), 268-273. doi: 10.3969/j.issn.1673-7202.2019.02.003

Tong, C. Y., Peng, M. J., and Shi, S. Y. (2018). Rapid identification of flavonoid compounds in pericarpium citri reticulatae by online extration high performance liquid chromatography diode array detection quadruple time of flight mass spectrometry. *Chinese journal of chromatography.* 36(3), 278-284. doi: 10.3724/SP.J.1123.2017.11031

Wang, G. W., Bao, B., Han, Z. Q., Han, Q. Y., and Yang, X. L. (2016). Metabolic profile of Fructus Gardeniae in human plasma and urine using ultra high-performance liquid chromatography coupled with high-resolution LTQ-orbitrap mass spectrometry. *Xenobiotica*. 46(10), 901-912. doi: 10.3109/00498254.2015.1132793

Wang, Y., Li, C. M., Huang, L., Liu, L., Guo, Y. L., Ma, L., et al. (2014). Rapid identification of traditional Chinese herbal medicine by direct analysis in real time (DART) mass spectrometry. *Anal. Chim. Acta.* 845, 70-76. doi: 10.1016/j.aca.2014.06.014

Xiang, H. J., Zhang, L. S., Song, J. N., Fan, B., Nie, Y. L., Bai, D., et al. (2016). The Profiling and Identification of the Absorbed Constituents and Metabolites of Guizhi Decoction in Rat Plasma and Urine by Rapid Resolution Liquid Chromatography Combined with Quadrupole-Time-of-Flight Mass Spectrometry. *Int. J. Mol. Sci.* 17(9), 1409. doi: 10.3390/ijms17091409

Xu, Y. Y., Cai, H., Cao, G., Duan, Y., Pei, K., Tu, S. C., et al. (2018). Profiling and analysis of multiple constituents in Baizhu Shaoyao San before and after processing by stir-frying using UHPLC/Q-TOF-MS/MS coupled with multivariate statistical analysis. *J. Chromatogr. B. Analyt. Technol. Biomed. Life. Sci.* 1083, 110-123. doi: 10.1016/j.jchromb.2018.03.003

Yang, N., Dong, Y. Q., Wu, M. F., Li, S. Z., Yu, H. X., and Yang, S. S. (2020). Establishing a rapid classification and identification method for the major triterpenoids of Alisma orientale. *Phytochem. Anal.* 31(3), 384-394. doi: 10.1002/pca.2907

Zhan, Z. L., Deng, A. P., Kang, L. P., Tang, J. F., Nan, T. G., Chen, T., et al. (2018). Chemical profiling in Moutan Cortex after sulfuring and desulfuring processes reveals further insights into the quality control of TCMs by nontargeted metabolomic analysis. *J. Pharm. Biomed. Anal.* 156, 340-348. doi: 10.1016/j.jpba.2018.04.045

Zhao, W. L., Huang, X. Q., Li, X. Y., Zhang, F. F., Chen, S. N., Ye, M., et al. (2015). Qualitative and Quantitative Analysis of Major Triterpenoids in Alismatis Rhizoma by High Performance Liquid Chromatography/Diode-Array Detector/Quadrupole-Time-of-Flight Mass Spectrometry and Ultra-Performance Liquid Chromatography/Triple Quadrupole Mass Spectrometry. *Molecules.* 20(8), 13958-13981. doi: 10.3390/molecules200813958

Zheng, G. D., Liu, M. S., Chao, Y. X., Yang, Y. H., Zhang, D. D., Tao, Y. W., et al. (2020). Identification of lipophilic components in Citri Reticulatae Pericarpium cultivars by supercritical CO_2_ fluid extraction with ultra-high-performance liquid chromatography-Q Exactive Orbitrap tandem mass spectrometry. *J. Sep. Sci.* 43(17), 3421-3440. doi: 10.1002/jssc.202000490

Zheng, Y. Y., Zeng, X., Peng, W., Wu, Z., and Su, W. W. (2019). Characterisation and classification of Citri Reticulatae Pericarpium varieties based on UHPLC-Q-TOF-MS/MS combined with multivariate statistical analyses. *Phytochem. Anal.* 30(3), 278-291. doi: 10.1002/pca.2812

Zhou, J. L., Liu, W., Guo, Z. X., and Chen, B. L. (2013). Fingerprint analysis of Fritillaria thunbergii using rapid resolution liquid chromatography coupled with electrospray ionization quadrupole time-of-flight tandem mass spectrometry. *China journal of chinese materia medica.* 38(17), 2832-2837. doi: 10.4268/cjcmm20131717

Zhou, J., Zhang, Y. J., Li, N., Zhao, D., Lu, Y., Wang, L. R., et al. (2020). A systematic metabolic pathway identification of Common Gardenia Fruit (Gardeniae Fructus) in mouse bile, plasma, urine and feces by HPLC-Q-TOF-MS/MS. *J. Chromatogr. B. Analyt. Technol. Biomed. Life. Sci.* 1145, 122100. doi: 10.1016/j.jchromb.2020.122100

Zhou, W., Shan, J. J., and Meng, M. X. (2018). A two-step ultra-high-performance liquid chromatography-quadrupole/time of flight mass spectrometry with mass defect filtering method for rapid identification of analogues from known components of different chemical structure types in Fructus Gardeniae-Fructus Forsythiae herb pair extract and in rat's blood. *J. Chromatogr. A.* 1563, 99-123. doi: 10.1016/j.chroma.2018.05.067
